# Environmental Selection Pressures Related to Iron Utilization Are Involved in the Loss of the Flavodoxin Gene from the Plant Genome

**DOI:** 10.1093/gbe/evv031

**Published:** 2015-02-16

**Authors:** Juan J. Pierella Karlusich, Romina D. Ceccoli, Martín Graña, Héctor Romero, Néstor Carrillo

**Affiliations:** ^1^Instituto de Biología Molecular y Celular de Rosario (IBR), CONICET-Universidad Nacional de Rosario, Ocampo y Esmeralda, Rosario, Argentina; ^2^Unidad de Bioinformática, Institut Pasteur Montevideo, Uruguay; ^3^Departamento de Ecología y Evolución, Facultad de Ciencias/CURE, Universidad de la República, Montevideo, Uruguay; ^4^Present address: Facultad de Ciencias Bioquímicas y Farmacéuticas, Universidad Nacional de Rosario; CONICET, Rosario, Argentina

**Keywords:** flavodoxin, ferredoxin, iron, photosynthesis, algae, cyanobacteria

## Abstract

Oxidative stress and iron limitation represent the grim side of life in an oxygen-rich atmosphere. The versatile electron transfer shuttle ferredoxin, an iron-sulfur protein, is particularly sensitive to these hardships, and its downregulation under adverse conditions severely compromises survival of phototrophs. Replacement of ferredoxin by a stress-resistant isofunctional carrier, flavin-containing flavodoxin, is a widespread strategy employed by photosynthetic microorganisms to overcome environmental adversities. The flavodoxin gene was lost in the course of plant evolution, but its reintroduction in transgenic plants confers increased tolerance to environmental stress and iron starvation, raising the question as to why a genetic asset with obvious adaptive value was not kept by natural selection. Phylogenetic analyses reveal that the evolutionary history of flavodoxin is intricate, with several horizontal gene transfer events between distant organisms, including *Eukarya*, *Bacteria*, and *Archaea*. The flavodoxin gene is unevenly distributed in most algal lineages, with flavodoxin-containing species being overrepresented in iron-limited regions and scarce or absent in iron-rich environments. Evaluation of cyanobacterial genomic and metagenomic data yielded essentially the same results, indicating that there was little selection pressure to retain flavodoxin in iron-rich coastal/freshwater phototrophs. Our results show a highly dynamic evolution pattern of flavodoxin tightly connected to the bioavailability of iron. Evidence presented here also indicates that the high concentration of iron in coastal and freshwater habitats may have facilitated the loss of flavodoxin in the freshwater ancestor of modern plants during the transition of photosynthetic organisms from the open oceans to the firm land.

## Introduction

Flavodoxins (Flds) are small (Mr = 16–20 kDa), soluble electron transfer proteins containing one mole of noncovalently bound flavin mononucleotide (FMN). They shuttle low-potential electrons between different donors and acceptors in redox-based metabolic pathways of a number of bacteria and algae (Sancho 2006; Pierella Karlusich et al. 2014). Fld activities largely match those of ferredoxins (Fds), ubiquitous redox carriers that utilize iron-sulfur (Fe-S) centers as prosthetic groups (Hanke and Mulo 2013). Fld and Fd do not share significant sequence or structural similarities (Ullmann et al. 2000), yet they can productively interact with essentially the same suite of redox partners, with Fld being usually less efficient (Carrillo and Ceccarelli 2003). The two proteins have been found in prokaryotes of all major taxa, in which they contribute reducing equivalents to sustain very different lifestyles (Zurbriggen et al. 2007).

Fds (and iron-sulfur proteins in general) were extensively used by the microorganisms thriving in the anaerobic environments of the primitive Earth, in which ferrous iron and sulfide were plentiful and readily available (Wächtershäuser 1992). They evolved in the absence of any selective pressure to avoid reactivity with oxygen or oxygen derivatives. Then, approximately 2,700 Ma, cyanobacteria developed oxygenic photosynthesis, and Fd was recruited into the photosynthetic electron transport chain of thylakoids to mediate electron transfer from photosystem I (PSI) to a number of metabolic, regulatory and dissipative pathways, including NADP^+^ reduction, and carbon, nitrogen and sulfur assimilation (Zurbriggen et al. 2008; Hanke and Mulo 2013). Oxygen levels remained low over the following 2 billion years or so, due to reaction with dissolved ferrous and sulfide ions, until their stocks became largely exhausted and atmospheric oxygen began to build up at the brink of the Precambrian (∼800 Ma). These biogeological changes gave rise to several new challenges on existing microorganisms, two of them directly affecting the utilization of Fe–S centers. First, oxidized iron precipitated in the form of ferric hydroxides and oxyhydroxides, drastically decreasing its bioavailability; and second, Fe–S clusters proved to be vulnerable (to various extents) to inactivation by oxygen and its partially reduced derivatives, the so-called reactive oxygen species, which were unavoidable byproducts of aerobic metabolism (Imlay 2006; Pierella Karlusich et al. 2014). Therefore, the high demand for Fe–S centers that aerobes inherited from their anaerobic ancestors did not suit well an oxygen-rich world. Different mechanisms had to be developed by cyanobacteria and other microorganisms to survive iron deficiency and oxidant toxicity. Among them, expression of Fld proved to be particularly useful as an adaptive resource to replace Fd, whose levels decline under iron limitation and oxidative stress (Erdner et al. 1999; Mazouni et al. 2003; Yousef et al. 2003; Singh et al. 2004; Chappell and Webb 2010; Thompson et al. 2011). In general, Fd is the normal electron carrier in phototrophs, whereas Fld expression is induced under various sources of environmental stress that result in Fd downregulation (Laudenbach et al. 1988; Erdner et al. 1999; Hagemann et al. 1999; Mazouni et al. 2003; Yousef et al. 2003; Singh et al. 2004; Chappell and Webb 2010; Thompson et al. 2011). Under such conditions, the flavoprotein takes over the functions of the iron-sulfur protein, allowing growth and reproduction of the organism, and permitting reallocation of the available iron to other demanding routes and enzymes.

Although Fld is found in all major algal taxa, it is absent from plants, even though plant Fd also declines under iron deprivation (Thimm et al. 2001) and many sources of environmental or oxidative stress (Zimmermann et al. 2004; Tognetti et al. 2006). Plants depend on alternative, multigenic strategies to cope with these hazards, such as optimization of iron uptake and induction of scavenging enzymes (Mittler et al. 2011; Kobayashi and Nishizawa 2012), without resorting to the substitutive responses found in photosynthetic prokaryotes and algae.

Genes that persist in an evolving genome and rise to fixation in a population are inferred to be functionally important and to enhance fitness. Conversely, in the absence of selection, it is expected that dispensable genes will be lost due to a mutational bias that favors deletion (Mira et al. 2001). Then, if tolerance to iron deficit was the critical imperative that determined the adaptive value of Fld expression, the most direct explanation for the disappearance of the Fld-coding gene from the plant genome could be that the flavoprotein no longer provided any adaptive advantage. For instance, ferroproteins other than Fd, with higher sensitivity to Fe limitation (or more critical to plant survival), could have appeared during the evolution of terrestrial plants, so that Fd replacement by Fld ceased to be of selective advantage. However, targeting of a cyanobacterial Fld to the chloroplasts of transgenic plants resulted in remarkable tolerance to oxidative stress, environmental challenges, and iron starvation (Tognetti et al. 2006, 2007; Coba de la Peña et al. 2010). The results indicate that the compensatory functions of Fld are still operating in higher plants in spite of the evolutionary divergence between the donor and host organisms. Moreover, studies on Fd-deficient lines transformed with an Fld-coding gene from cyanobacteria showed that the development of the tolerant phenotype relied, at least partially, on substitution of the activities of indigenous Fd counterparts as in microorganisms (Blanco et al. 2011). Then, the question remains open as to why Fld was lost during the evolution of terrestrial plants.

We address herein this question using the tools of evolutionary and ecological genomics to analyze public domain genetic, genomic, and metagenomic data sets. We constructed a detailed phylogenetic tree of available Fld sequences, which shows that they form a monophyletic group, and compared Fld evolutionary history with those of essential photosynthetic proteins such as isofunctional Fd and the manganese-stabilizing protein PsbO, a core subunit of photosystem II (PSII). The results suggest that the gene encoding Fld might have been transferred several times by horizontal gene transfer (HGT) between different domains of life, both photosynthetic and nonphotosynthetic, illustrating the versatility of this electron shuttle as an adaptive resource to face certain environmental onslaughts. Although Fld-coding genes are present in all major algal phyla, closely related organisms may or may not contain Fld depending on their habitats. Fld-lacking species are overrepresented in iron-replete freshwater and coastal regions. Evaluation of cyanobacterial genomes and metagenomic data obtained from various locations also confirmed a bias against Fld-containing organisms in Fe-rich environments. Taken together, the results indicate that there was little or no selection pressure to retain Fld in neritic algae, and that loss of the Fld-coding gene from the plant genome might be related to ecological adaptations to iron bioavailability and the successive stages of land colonization (Zurbriggen et al. 2007). Terrestrial plants evolved from coastal/freshwater macroalgae which thrived in an environment where iron was both abundant and readily accessible, strongly suggesting that the founder algal species from which terrestrial plants originated already lacked Fld.

## Materials and Methods

### Database Search

BLAST (“tBLASTn” and “BLASTp” programs) searches (Altschul et al. 1997) were conducted against DNA and protein sequences available at the Integrated Microbial Genome (IMG) (http://img.jgi.doe.gov, last accessed March 3, 2015; Markowitz et al. 2012), the National Center for Biotechnology Information (NCBI) (http://www.ncbi.nlm.nih.gov, last accessed March 3, 2015), the DOE Joint Genome Institute (http://genome.jgi-psf.org, last accessed March 3, 2015), the Taxonomically Broad EST (expressed sequence tag) Database (TBestDB; http://tbestdb.bcm.umontreal.ca/, last accessed March 3, 2015; O’Brien et al. 2007) and the sequenced genomes and transcriptomes listed in supplementary table S1, Supplementary Material online. All searches were performed over the February–March 2014 versions of the databases.

The Fd and PsbO protein sequences from *Prochlorococcus marinus* MED4, and the Fld sequences from *Chondrus crispus*, *P. marinus marinus* CCMP1375, *Trichodesmium erythraeum* IMS101, *Escherichia coli*, *Azorhizobium caulinodans* ORS571, *Azoarcus* sp. BH72, *Sulfurospirillum deleyianum* DSM6946, *Sulfuricurvum kujiense* YK-1, and *Pelobacter carbinolicus* DSM2380 were used as queries for the “tBLASTn” and “BLASTp” searches to identify the homologous genes in different organisms with an e^−^^5^ *e* value threshold.

### Profile Searches

Homologous sequences with solved structures were searched using profile hidden Markov models, with the HHsearch suite (Söding 2005). Briefly, a diverse set of 63 sequences obtained with “BLASTp” were aligned with T-Coffee (Notredame et al. 2000) and used as a search profile against the Protein Data Bank. As a test control, predicted protein sequence XP_002291468.1 from *Thalassiosira pseudonana* was used as query within the entire pipeline of the HHpred server (Söding 2005).

### Sequence Alignment and Phylogenetic Analysis

**Fig. 1.—:**
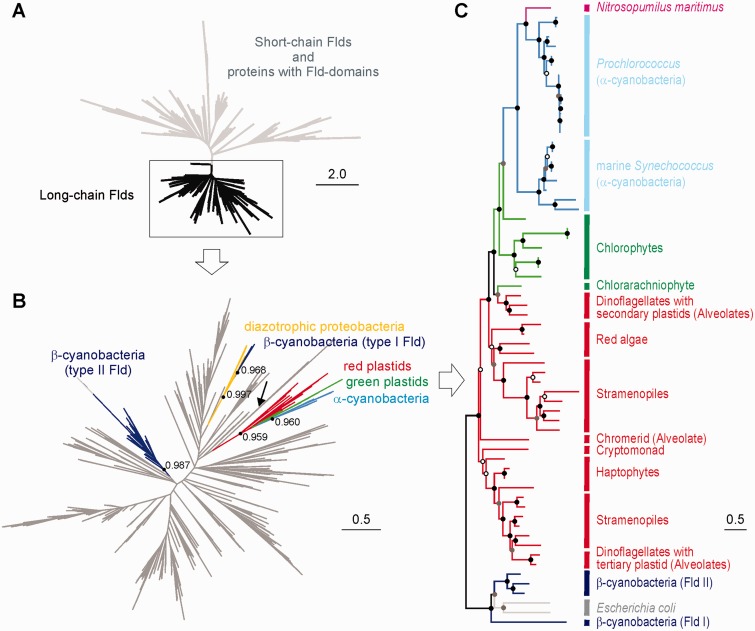
Phylogenetic relationships among Fld protein sequences. (*A*) Phylogenetic tree of Flds and proteins with Fld-like domains. The tree topology indicates that short-chain Flds and Fld-like domains are phylogenetically close to each other, and that long-chain Flds (square) have a monophyletic origin. (*B*) Phylogeny of long-chain Flds, amplified from the squared region of figure 1*A*, showing the split between β-cyanobacterial and eukaryotic/α-cyanobacterial clades. Gray branches correspond to nonphotosynthetic microorganisms. The root position (indicated by an arrow) was inferred using short-chain Fld sequences. The aLRT values are shown for the clades of interest: Eukaryotic/α-cyanobacterial clade, α-cyanobacterial clade, type II β-cyanobacterial Fld clade, type I β-cyanobacterial Fld clade, diazotrophic proteobacterial/type I β-cyanobacterial Fld clade. (*C*) Phylogenetic relationship among eukaryotic Flds. The tree was rooted using as outgroups the long-chain Flds from *E. coli* and the β-cyanobacteria *Trichodesmium erythraeum* IMS101*, Anabaena variabilis* ATCC29413, and *Anabaena cylindrica* PCC7122. Nodes annotated with solid circles have 0.75 aLRT values or greater, those with shaded circles have 0.50–0.75 aLRT values, and those with hollow circles have less than 0.50 aLRT values. The scale bars indicate the number of expected amino acid substitutions per site per unit of branch length.

In some cases, CDHIT (Li and Godzik 2006) was used to reduce redundancy within each protein set before sequence alignment and phylogenetic analysis in order to facilitate the subsequent tree interpretation and to reduce taxonomic bias. In the case of Fld, the tree displayed in figure 1*A* was constructed after applying a CDHIT cut-off value of 70% identity to the whole sequence set retrieved as described above. The long-chain Fld tree (fig. 1*B*) contains the reduced set of corresponding sequences using a 90% identity threshold with CDHIT.

Protein sequences were aligned with MAFFT version 6 using the G-INS-I strategy (Katoh and Toh 2008). In addition, a set of 11 X-ray Fld structures solved with bound FMN was selected for structural analysis. Pairwise identities of these proteins ranged between 21% and 69%. Multiple structure alignments were computed with CE (Shindyalov and Bourne 1998), MUSTANG (Konagurthu et al. 2006), and Stamp (Russell and Barton 1992). The resulting multiple sequence alignment was used as a template for producing an extra alignment of the Fld sequences. All methods of structural superposition agreed in the conserved structural regions. Despite low pairwise sequence identities, the root mean square deviation of the crystal structures was remarkably low (<3 Å over ∼160 residues). The diverse structures analyzed possess equivalent binding sites, with key structural and functional residues retained by evolution (supplementary fig. S1, Supplementary Material online). Interestingly, phylogenetic analyses yielded essentially the same results even when omitting the structural information to perform the alignments, hence the simplest procedure is the one presented in the Results section. Phylogenetic trees were generated using the LG substitution model in PhyML 3.0 (Guindon et al. 2010). Four categories of rate variation were used. The starting tree was a BIONJ tree and the type of tree improvement was subtree pruning and regrafting. Branch support was calculated using the approximate likelihood ratio test (aLRT) with a Shimodaira–Hasegawa-like (SH-like) procedure.

**Fig. 2.—:**
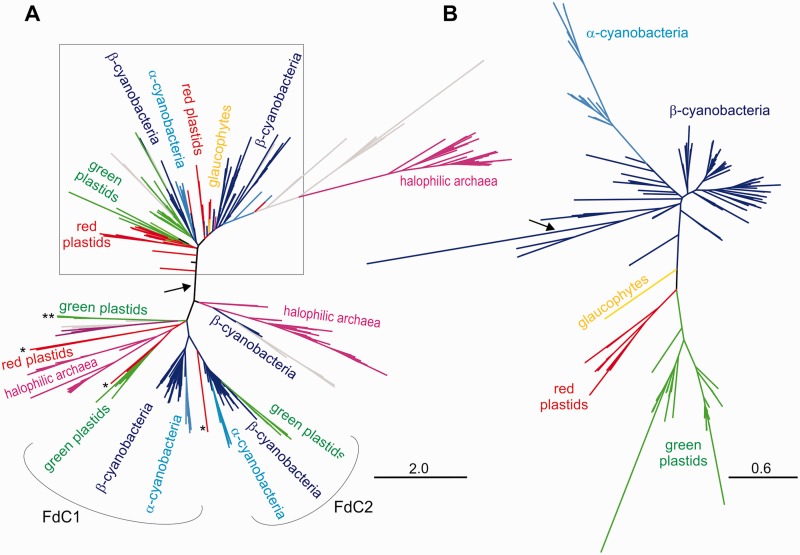
Phylogenetic trees of photosynthetic Fd (*A*) and PsbO (*B*) protein sequences. (*A*) The squared region in the Fd tree includes photosynthetic Fds, the Fds from plant roots (in the “green plastids” lineages) and non-photosynthetic alveolates (in the “red plastids” lineages), and the Fds involved in electron donation to nitrogenase in diazotrophic β-cyanobacteria. More divergent Fd isoforms, such as FdC1 and FdC2 from plants, green algae and cyanobacteria, Fdx3 from the green alga *C. reinhardtii* (“green plastids” lineage indicated with **), the nonphotosynthetic Fds from the brown alga *Ectocarpus siliculosus* (“red plastids” lineages marked with *), and the Fds from halophilic archaea are located outside the squared region. A few proteobacteria and actinobacteria have Fds and proteins with Fd-like domains that appear scattered in different parts of the tree (gray lineages). The arrow indicates the root, as transferred from the tree described in supplementary figure S4, Supplementary Material online. (*B*) The phylogenetic tree of PsbO illustrates the succession of endosymbiotic events during the evolution of plastids. The root position (indicated by an arrow) was determined using *Gloeobacter violaceous* sequence as outgroup. The scale bar indicates the number of expected amino acid substitutions per site per unit of branch length.

For evaluating the Fd phylogeny, the set of sequences retrieved as described above were reduced using a 40% identity threshold with CDHIT, and a phylogenetic tree was constructed (supplementary fig. S4, Supplementary Material online), in which the clade containing the cyanobacterial/plastidic Fds was identified. The tree of figure 2*A* contains the proteins whose BLAST best hit sequences grouped in the cyanobacterial/plastidic Fd clade of the previous tree. To minimize taxonomic bias and reduce the complexity of the tree, a cut-off value of 80% identity using CDHIT was applied to obtain the Fd phylogeny of figure 2*A*.

### Evaluation of the Distribution of Fld-Coding Genes in Different Photosynthetic Organisms and Habitats

The presence or absence of Fld-coding genes in different photosynthetic organisms was determined by BLAST searches in sequence databases as described above, and by bibliography surveys. To minimize false-negative cases, only completely sequenced genomes were considered for establishing the absence of Fld-coding genes.

The isolation sites of the analyzed organisms were retrieved from the literature. To establish the phylogenetic relationships between the selected organisms, a maximum-likelihood tree was inferred from their 18S rRNA sequences in algae and from their 16S rRNA in cyanobacteria. All aligned 18S and 16S rRNA sequences were retrieved from the SILVA project (http://arb-silva.de; Quast et al. 2013), and phylogenetic analyses were performed using PHYML v3.0 with default parameters. In those algal species in which the 18S rRNA sequence was not available, the sequence belonging to another member of the same genus was included (supplementary table S1, Supplementary Material online).

Phylogenetic trees with added circles and bar charts showing presence or absence of Fld and iron availability of each sample, respectively, were visualized using the iTOL web server (http://itol.embl.de/; Letunic and Bork 2007).

### Metagenomic Analysis

The abundance of Fld-coding genes from photosynthetic organisms was analyzed in the metagenomic data from samples collected within a 0.1–0.8 µm size range during the Global Ocean Sampling (GOS) expedition (Rusch et al. 2007). Metagenomes were downloaded from the CAMERA database (Seshadri et al. 2007). The Sargasso Sea sample GS000A was not used in the analysis due to the suspicion of contamination (DeLong 2005). BLAST searches were conducted using a diverse set of Fld protein sequences with an *e* value of e^−^^10^ as threshold. Then, for taxonomic assignment, phylogenetic placement of a validation set of 20 recruited reads was performed on the reference Fld phylogenetic tree. The results were, to our purposes, identical to assigning the best hit against NCBI’s nr database using “BLASTx”; hence, this latter procedure was used. To estimate the frequency of genes in each cyanobacterial genus at each geographic location, the number of Fld-like hits was divided by the average number of hits with sequence similarity to single-copy core genes found at each site. The core genes chosen were those encoding recombinase A (RecA), the RNA polymerase β subunit (RpoB), the DNA gyrase B subunit (GyrB), and the PSII core subunit PsbO. The numbers of hits observed for a particular gene were normalized to the mean length of the query gene in kilobases.

### Statistical Analyses

The Generalized Estimating Equations (GEE) approach implemented in the APE package (Paradis et al. 2004) was used to evaluate the possible correlation between iron bioavailability and presence of the Fld-coding gene. GEE incorporates a phylogenetic distance matrix into the framework of a general linear model. The matrices were obtained from the maximum-likelihood trees inferred from the 18S and 16S rRNA sequences in the case of algae and cyanobacteria, respectively. The presence/absence of the Fld-coding gene was considered as the response variable and the Fe level as the explanatory variable. A binomial distribution (with logistic link function) was assumed as the response, given the discrete nature of the data. When two or more species show an rRNA sequence divergence lower than 0.5% and the same pattern of Fld presence/absence and Fe availability, only one was considered for the GEE analysis to decrease any bias in favor of related species with high numbers of isolations (*n* = 78 for algae, *n* = 118 for cyanobacteria).

## Results

### Paralogy and Genome Location of Genes Encoding Fld in Phototrophs

In photosynthetic eukaryotes, Fld is only found in plastids. These organelles originated by a single endosymbiotic event of an ancient cyanobacterium in the common ancestor of glaucophytes, red algae, and green algae (supplementary fig. S2, Supplementary Material online, see also Keeling 2013). The three lineages originating from this primary endosymbiotic event only represent a fraction of the eukaryotic phototrophs, as most extant algae acquired their plastids through secondary endosymbioses of a green or red alga. Green algae were involved in two independent secondary endosymbioses which led to euglenids and chlorarachniophytes (Rogers, Gilson, et al. 2007). Red algae, instead, appear to have been successfully taken up only once, giving rise to a diversified group called chromalveolates, consisting of haptophytes, cryptophytes, stramenopiles, and alveolates (supplementary fig. S2, Supplementary Material online). Alveolates, in turn, comprise dinoflagellates, which include both photosynthetic and nonphotosynthetic species, apicomplexans, most of which lack photosynthesis, and nonplastid-bearing ciliates (Keeling 2013). Therefore, although all plastids are closely related, the algae containing them may not be.

In all cases described so far, Fld is nuclear-encoded with a plastid-targeting sequence. No Fld-coding genes have been retrieved from the 82 fully sequenced algal plastomes listed in the NCBI Organelle Genome Resources (www.ncbi.nlm.nih.gov/genomes/ORGANELLES/organelles.html), or from the five sequenced nucleomorphs, which are remnants of the secondary endosymbiont nuclei still retained in cryptophytes and chlorarachniophytes (Moore et al. 2012). Therefore, the gene encoding Fld must have been transmitted into the host nucleus several times during the successive endosymbiotic events (Keeling 2013).

In most studied eukaryotic organisms containing Fld, its gene is present in one copy. Exceptions are some stramenopiles such as diatoms and pelagophytes, which generally have two copies, only one of them regulated by iron bioavailability (Whitney et al. 2011).

With respect to cyanobacteria, they have been divided in α- and β-cyanobacteria, as they hold form IA rubisco and α-carboxisomes, or form IB rubisco and β-carboxisomes, respectively (Rae et al. 2013). Although β-cyanobacteria form a paraphyletic group, α-cyanobacteria are monophyletic and consist mainly of picocyanobacterial members of the *Synechococcus* and *Prochlorococcus* genera, which are the dominant species in marine habitats (Coelho et al. 2013).

The gene copy number of Fld-containing cyanobacteria is also variable. Out of 107 completely sequenced genomes containing Fld-coding genes, 76 of them possess a single copy, 25 harbor two copies, and 6 have more than two copies. Almost all Fld-containing α-cyanobacteria possess only one gene copy, whereas β-cyanobacteria can contain two or more isoforms.

### Fld Phylogeny

Flds are classified as short-chain or long-chain depending on the presence of a 20-amino acid loop of unknown function, with the photosynthetic Flds belonging to the latter class (Sancho 2006). Fld trees have been reported previously (Lin et al. 2009; Whitney et al. 2011; Burki et al. 2012; Pérez-Dorado et al. 2013). However, they were constructed with specific purposes and therefore provided a partial representation of Fld phylogeny. We have herein included many more sequences to have a broader set of organisms, using Flds identified by sequence similarity search methods across all genomes of bacteria, archaea, and eukaryotes available at the IMG database, as well as eukaryotic Fld sequences retrieved from other databases (fig. 1, see Materials and Methods). Our results indicate that long-chain Flds form a monophyletic group distributed between photosynthetic eukaryotes and various bacterial phyla such as *Cyanobacteria*, *Proteobacteria*, *Bacteroidetes*, *Chlorobi*, *Spirochaetes*, *Tenericutes*, *Chloroflexi*, *Acidobacteria*, *Actinobacteria,* and *Fusobacteria* (fig. 1*A*).

Surprisingly, the Flds from photosynthetic organisms do not group together within the long-chain Fld tree, but are divided among three different clades (fig. 1*B*). The eukaryotic and α-cyanobacterial sequences assemble into a single clade, whereas the Flds from β-cyanobacteria are split into two separate clades, named type I and II (Lin et al. 2009). Type II β-cyanobacterial Flds form a highly supported clade (aLRT value 0.987), and their expression is enhanced by iron deficiency (Chappell and Webb 2010). In contrast, type I Flds do not respond to iron limitation and are only found in some diazotrophic β-cyanobacteria. They are proposed to act as electron donors to nitrogenase in these organisms (Saito et al. 2011), and group with the Flds of some nonphotosynthetic diazotrophic proteobacteria (fig. 1*B*, aLRT value 0.997), suggesting that they were acquired from proteobacteria by HGT (Lin et al. 2009).

The algal sequences form a specific and strongly supported clade with the α-cyanobacterial Flds. The aLRT values were 0.959 and 0.99 for the trees displayed in figure 1*B* and *C*, respectively, which is very significant considering the size of the Fld protein. The eukaryotic sequences divide themselves into two branches (fig. 1*C*). One of them contains most chromalveolate sequences. The evolutionary trajectory of the other branch is complex. Two successive divisions give origin to three distinct Fld lineages: 1) Red algae/stramenopiles (the iron-responsive isoform of diatoms and pelagophytes), 2) chlorarachniophytes/secondary-plastid bearing dinoflagellates, and 3) green algae (fig. 1*C*). According to the tree topology, three Fld-coding gene copies could have been present in the ancient red alga that was supposedly engulfed by a heterotrophic protist to give rise to chromalveolates. One or more copies may have been differentially lost afterwards.

Within the *Eukarya*/*α-Cyanobacteria* clade, the eukaryotic Flds are basal in relation to their α-cyanobacterial counterparts (fig. 1*C*). This topology differs from those inferred from 16S rRNA or PsbO (see next section), which show all cyanobacterial sequences grouping in a basal position with respect to the eukaryotic clades, in agreement with the existence of the primary endosymbiotic event. The conflicting topology involving the α-cyanobacterial position in the Fld protein tree with respect to the species-plastid tree might indicate an HGT event from an eukaryotic donor to an α-cyanobacterium. Within this context, the alignment of the Fld sequences shows a conserved insertion present in the Flds from eukaryotes and α-cyanobacteria, but not in those from the remaining bacteria (squared region in supplementary fig. S3, Supplementary Material online). Moreover, in an Fld phylogenetic tree restricted to α-cyanobacteria and primary plastid-bearing algae (red and green algae), the path in the tree connecting these two groups of organisms represents just approximately 3% of the total sum of evolutionary distances of the tree, whereas this value rises to approximately 30% in the equivalent tree of PsbO (data not shown), suggesting either a very idiosyncratic rate of change or, most likely, a more recent divergence. Given the strong support for the existence of the clade comprising *Eukarya* and α-cyanobacteria (and an archaea, see below), and the presence of the same insertion in the sequences of all members of the clade (supplementary fig. S3, Supplementary Material online), it is highly unlikely that this particular topology results from phylogenetic reconstruction artifacts. Instead, it provides clues to understand the evolutionary processes involving the trafficking of the Fld gene among these groups of organisms.

The phylogenetic tree of Fld shows still another putative HGT event, in this case from an α-cyanobacterium to the nonphotosynthetic marine archaea *Nitrosopumilus maritimus*, one of the most abundant microorganisms in the global oceans, which can grow autotrophically through ammonium oxidation. In fact, extensive HGT might have occurred from α-cyanobacteria to this archaea (Walker et al. 2010).

In conclusion, the Fld tree topology indicates that this flavoprotein has a complex evolutionary history characterized by differential losses and duplications, both in prokaryotes and eukaryotes, and by HGT events between organisms belonging to different domains of life and displaying completely different lifestyles.

### Comparative Phylogeny of Fd and PsbO

In order to determine whether the described features of the Fld tree are specific of this flavoprotein or represent more general characteristics of photosynthetic proteins, we compared its phylogeny with those of essential components of oxygenic photosynthesis such as the isofunctional electron carrier Fd and the PSII core subunit PsbO (fig. 2). Unlike Fld (and PsbO, see below), Fd could be plastid- or nuclear-encoded depending on the species (Lommer et al. 2010). Although in the green-plastid lineage algae the Fd-coding gene is always located in the nuclear genome, in the glaucophyte *Cyanophora paradoxa* and in the vast majority of the red-plastid lineage algae it is found in the plastome. The number of different Fd-coding genes present in the genome also varies among species: Land plants, green algae, and cyanobacteria contain two or more Fds, whereas algae from the red-plastid lineage and *C. paradoxa* usually have only one isoform.

All described Fd and Fd-like domain sequences group together in a general phylogenetic tree (supplementary fig. S4, Supplementary Material online), suggesting a monophyletic origin for photosynthetic and nonphotosynthetic Fds. However, a complete Fd tree displays great complexity (supplementary fig. S4, Supplementary Material online). We therefore set a stringent criterion to retrieve those sequences more closely related to the Fds from cyanobacteria and plastids (see Materials and Methods). This procedure excluded the Fds present in mitochondria and most heterotrophic microorganisms, as well as most Fd-like domains of multidomain enzymes. The resulting “photosynthetic Fd” tree (fig. 2*A*) harbors the Fds not only from plastids and cyanobacteria but also from halophilic archaea and a few proteobacteria and actinobacteria. Although simplified, the evolutionary history of these Fds is still complicated by the high degree of paralogy and functional specialization of the protein.

Fds contained in the reduced tree of figure 2*A* split into two major clusters. Contrary to the Fld phylogeny (fig. 1), all canonical Fds from photosynthetic organisms group together, including those present in nonphotosynthetic plastids from plants and alveolates, and the Fd isoforms responsible for electron donation to nitrogenase in diazotrophic β-cyanobacteria (squared region in fig. 2*A*). The FdC1 and FdC2 proteins from plants, green algae, and cyanobacteria, which are more divergent Fd forms of unknown function, constitute the bulk of the second cluster, which also contains the Fdx3 isoform of the green alga *Chlamydomonas reinhardtii* and nonphotosynthetic Fds from brown algae belonging to the red-plastid lineage (labeled with asterisks in fig. 2*A*). As in the case of Fld, the “photosynthetic Fd” tree is plagued with putative interdomain HGT events. Some of them involve gene transfers between a cyanobacterium or alga and an archaea belonging to the *Halobacteriaceae* (Nelson-Sathi et al. 2012), although the direction of the HGT event cannot be inferred due to the complexity of the tree. Most members of this halophilic archaeal family encode at least two Fd isoforms. One of them donates electrons to nitrate reductase, nitrite reductase, and glutamate synthase (Pire et al. 2014), and is phylogenetically closer to the typical Fds of plastids and cyanobacteria (the branch stemming from the squared region of fig. 2*A*). The function(s) of the other isoforms has not been yet established, but they are phylogenetically more related to FdC1/FdC2 (fig. 2*A*). Other putative HGT events may have occurred between cyanobacteria/algae and proteobacteria/actinobacteria, as previously reported by Sjöblom et al. (2008) and Grinter et al. (2012). These sequences appear scattered throughout the tree (gray lineages in fig. 2*A*). Once again, the basal nature of cyanobacterial or eukaryotic Fd sequences cannot be decided due to the intrinsic complexity of the tree.

With respect to PsbO, this protein presents several advantages to study the evolutionary relationships among plastids and cyanobacteria. First, it is unique to organisms that carry out oxygenic photosynthesis. Second, being nuclear-encoded, phylogenetic analyses are not affected by the different evolutionary rates of the nuclear and plastid genomes (Wolfe et al. 1987). Finally, its high sequence conservation allows the analysis of relationships over large evolutionary distances. The *psbO* gene is single-copy in the vast majority of cyanobacteria and algae, but has several copies in land plants.

The phylogeny of PsbO reflects a simpler evolutionary history than those of Fld and Fd (fig. 2*B*), and is consistent with those of other photosynthetic proteins such as PsbA (Moore et al. 2008), and with the 16S rRNA sequences (Janouškovec et al. 2012). The succession of endosymbiotic events that occurred during the evolution of algae is clearly illustrated by the phylogenetic tree of PsbO. In contrast to the Fld phylogeny, the PsbO sequences from α- and β-cyanobacteria group together, in a basal position with respect to the eukaryotic sequences (fig. 2*B*). In turn, the eukaryotic sequences exhibit three clades: The glaucophytes clade, the red-plastid lineage clade (red algae and chromalveolates), and the green-plastid lineage clade (green algae, land plants, euglenids, and chlorarachniophytes).

### Fld Is Absent from Individual Species from Most Algal Lineages

Many reports over the years have indicated that Fld-coding genes are ubiquitous but not universal in cyanobacteria and algae (Mazouni et al. 2003; Rocap et al. 2003; Pankowski and McMinn 2009; Desai et al. 2012). These observations suggest that Fld might represent a nonessential trait of adaptive value under certain hostile situations, as opposed to those genes whose products play essential roles in the cell and are therefore conserved in all species within a given lineage. Following the hypothesis that Fld loss from the plant genome was a consequence of the evolutionary history of *Viridiplantae* (Zurbriggen et al. 2007), we deemed it necessary to first determine how extensively the gene pervades the cyanobacterial and algal taxa, and the phylogenetic relationships existing between Fld-containing and Fld-lacking photosynthetic organisms.

**Fig. 3.—:**
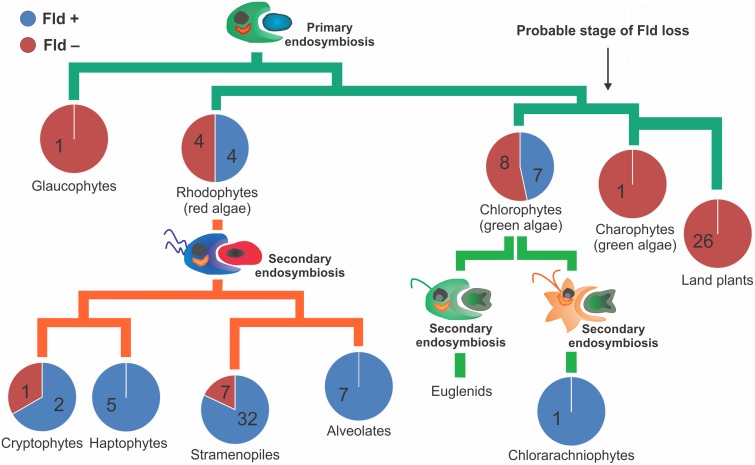
Phylogenetic distribution of Fld in photosynthetic eukaryotes. Numbers within each circle indicate those species containing (blue) or not (purple) Fld-coding genes. The data suggest that the Fld-coding gene might have been lost during the split between chlorophytes and streptophytes (indicated by an arrow).

Fld-coding genes were found across all major algal lineages including green and red algae, as well as those groups derived from secondary and tertiary endosymbioses (fig. 3). Notable exceptions are glaucophytes and euglenids, although this observation should be taken with caution, because only one sequenced genome is available from the former (*C. paradoxa*), and none from the latter. On the other hand, analysis of genome databases confirmed the absence of this flavoprotein in a number of algal species belonging to all major lineages (fig. 3, supplementary table S1, Supplementary Material online), except for chlorarachniophytes, haptophytes, and alveolates. In the case of chlorarachniophytes, only the *Bigelowiella natans* genome is available. Although all sequenced genomes from the two other taxa have been shown to contain one or more copies of the Fld-coding gene, Fld absence has been reported in some species using immunological methods under both iron-replete and iron-deficient conditions (Erdner et al. 1999). To minimize false negatives, we only worked with completely sequenced genomes.

The presence of Fld was detected in four red algal species, as well as in organisms resulting from subsequent endosymbiotic events involving red algae: 32 stramenopiles, 5 haptophytes, 2 cryptophytes, and 7 alveolates. Absence of Fld was confirmed in 4 red algae, 1 cryptophyte, and 7 stramenopiles (fig. 3).

Within the *Viridiplantae*, all the organisms in which the presence of Fld was confirmed belong to the chlorophytes (7 species), although it was absent from eight other species of the phylum (fig. 3). As indicated, Fld has also been detected in a chlorarachniophyte, *B. natans*, whose plastids derive from secondary endosymbiosis of a chlorophyte (Rogers, Gilson, et al. 2007). The flavoprotein has not been found in streptophytes, which consist of land plants (embryophytes) and charophytes, a group of freshwater algae that represent the sister lineage of land plants (fig. 3), and from which plants are proposed to have evolved (Leliaert et al. 2011). The recently released genome of the charophyte *Klebsormidium flaccidum* does not contain Fld-coding genes (Hori et al. 2014), and no Fld sequences could be retrieved from the EST collections of eight species from the six distinct groups of these algae (Timme et al. 2012). Finally, the absence of Fld-coding genes in the genomes of land plants was confirmed in all fully sequenced genomes available at the NCBI (http://www.ncbi.nlm.nih.gov/genomes/PLANTS/PlantList.html) and the IMG databases. They include the lycophyte *Selaginella moellendorffii*, a “primitive” vascular plant, as well as the moss *Physcomitrella patens*, a bryophyte, phylogenetically closer to the first green algal lineage that successfully colonized terrestrial habitats. Thus, the distribution of the gene encoding Fld implies that it has been most likely lost during the split between chlorophytes and streptophytes (indicated by an arrow in fig. 3), long before the origin of plants.

### Environmental Distribution of Fld-Coding Genes from Algae

**Fig. 4.—:**
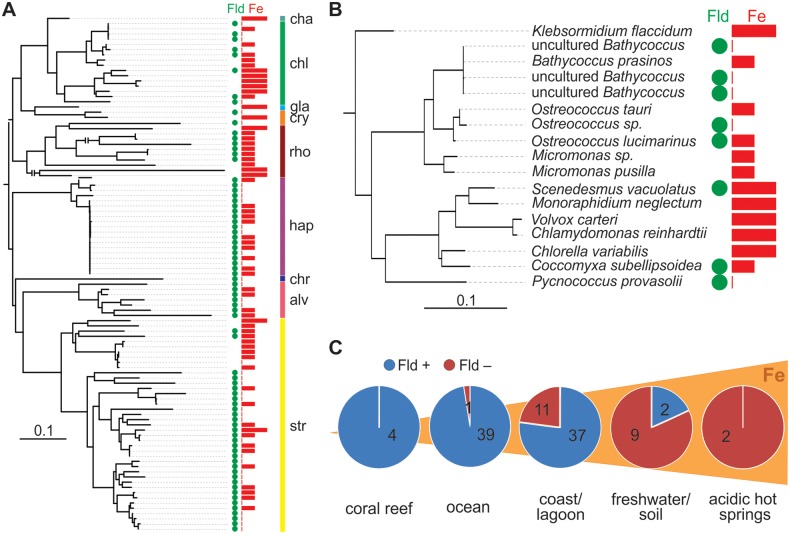
Environmental and phylogenetic distribution of Fld-coding genes among eukaryotes. (*A*) Phylogenetic tree of photosynthetic eukaryotes based on the 18S rRNA sequences. For each species, the presence (green circles) or absence of Fld, as well as the iron level (red bars), at the site of isolation is shown. Length of the red bars provides an estimation of iron level as high, intermediate, or low. The GEE statistical analysis, which takes into account the phylogenetic relationships of the organisms under study, shows a strong negative correlation (*P* < 10^−5^) between the presence of the Fld-coding gene and iron bioavailability. The tree was rooted between primary plastid-bearing algae and those whose plastids derived from secondary and subsequent endosymbiotic events. The scale bar indicates the number of substitutions per site. Two branches were shortened and interrupted by a double line. alv, alveolates; chr, chlorarachniophytes, cha, charophytes; chl, chlorophytes; cry, cryptophytes; gla, glaucophytes; hap, haptophytes; rho, rhodophytes (red algae); str, stramenopiles. (*B*) The region of the phylogenetic tree corresponding to chlorophytes and the charophyte *K. flaccidum* is shown in the enlarged view. (*C*) Distribution of Flds from eukaryotic phototrophs in different habitats. Fld presence (blue) or absence (purple) is represented by color codes, whereas the total numbers of isolates analyzed are indicated within each circle. The pie charts are only intended to be a visual aid as they do not show the phylogenetic relationships between isolates, and the numbers of isolates vary widely among habitats. The species/strains isolated from each habitat, their phylum/class classification, and the presence/absence of the Fld-coding gene can be found in supplementary table S1, Supplementary Material online. Increasing iron levels are intended for illustration and do not represent actual concentrations.

We investigated the association between Fld presence/absence and environmental iron levels, taking into consideration the phylogenetic relationships between the organisms analyzed. For this purpose, we constructed a maximum-likelihood tree inferred from their 18S rRNA sequences. The phylogenies shown in figure 4*A* and *B* are coherent with previous publications (Moon-van der Staay et al. 2001). Then, the iron levels in the site of isolation were recorded (supplementary table S1, Supplementary Material online). Each environment has complex chemistry and physics but to simplify the analysis it is considered that iron tends to increase in coastal, freshwater, and acidic/neutral hydrothermal habitats (fig. 4*C*), due to suspended sediments and aerial inputs from land (Jickells et al. 2005). In contrast, iron deficiency is predicted in as much as 40% of the open ocean, notably in the Southern Ocean as well as in the equatorial and north Pacific (Moore et al. 2001). Fe is abundant in firm land, but its bioavailability is compromised by low solubility, especially in alkaline calcareous soils (Guerinot and Yi 1994).

Fld-containing organisms have been isolated from different habitats, but with a clear bias toward iron-deficient environments such as open oceans (fig. 4*A* and supplementary fig. S5, Supplementary Material online). Conversely, all but one of the 23 cases of Fld absence corresponded to species from iron-rich environments, including members of both the green- and red-plastid lineages (supplementary table S1, Supplementary Material online). As indicated previously, no Fld sequence could be retrieved from the genomic or EST databases for organisms belonging to the charophytes, which are found in freshwater environments.

Closely related organisms may or may not contain Fld depending on their habitats. This situation is very clear in the chlorophytes *Bathycoccus* and *Ostreococcus* (fig. 4*B*)*.* Both are members of the order *Mamiellales*, which is the most abundant group of green-plastid lineage algae in marine habitats. Fld was absent from the complete genome sequence of *B. prasinos* strain RCC1105 isolated from the coast of the Mediterranean Sea (Moreau et al. 2012). However, the gene was found in three metagenome samples from cells sorted by flow cytometry and assigned to *Bathycoccus*, one collected from the deep chlorophyll maximum (DCM) in the tropical Atlantic (Monier et al. 2012), and the remaining two from the Pacific upwelling off the central coast of Chile at different depths, 5 and 30 m (Vaulot et al. 2012). The coast of the Mediterranean Sea is an iron-rich region, the tropical Atlantic Ocean suffers from chronic Fe deficiency, and the region off central Chile represents an intermediate situation.

Likewise, the three sequenced species of *Ostreococcus* are from contrasting environments (fig. 4*B***)**. Fld is present in *O. lucimarinus*, a surface-isolated strain from the coastal area of the Pacific Ocean (Palenik et al. 2007), and in *Ostreococcus sp*. RCC809, a low-light adapted strain isolated from the DCM (105 m depth) in the tropical Atlantic Ocean (http://genome.jgi-psf.org/OstRCC809_2). In contrast, Fld is absent from *O. tauri*, which was isolated from the shallow (4-m mean depth) and eutrophic Thau lagoon in the Mediterranean coast of France (Derelle et al. 2006).

In order to test the association between the presence of Fld-coding genes and iron bioavailability, we used a GEE approach which takes into account the phylogenetic relationships of the organisms under study (Paradis and Claude 2002). This is a crucial point as species cannot be regarded as independent observations because they are linked by their phylogenetic relationships. Closely related species with long shared evolutionary history may be more similar in many characteristics compared with more distantly related species, and the strength of the correlation between Fld absence/presence and Fe levels will depend on the phylogenetic distance among the organisms under study. GEE incorporates a phylogenetic distance matrix, derived from the phylogenetic trees of the relevant organisms, into the framework of a general linear model. In the case of algae, this matrix was obtained from the maximum-likelihood tree inferred from the 18S rRNA sequences (fig. 4*A*). The GEE analysis shows a strong negative correlation (*P* < 10^−^^5^) between the presence of the Fld-coding gene and iron bioavailability (defined as scarce in open ocean, medium in coastal environments, and high in freshwater habitats).

These observations suggest that selection pressure to retain Fld may be negligible in algae dwelling in iron-replete environments such as freshwater and coast (fig. 4*C*), and endorse the hypothesis that the absence of an Fld-coding gene in the plant genome may be tracked to its loss from the algal precursor of land plants.

### Environmental Distribution of Fld-Coding Genes from Cyanobacteria

**Fig. 5.—:**
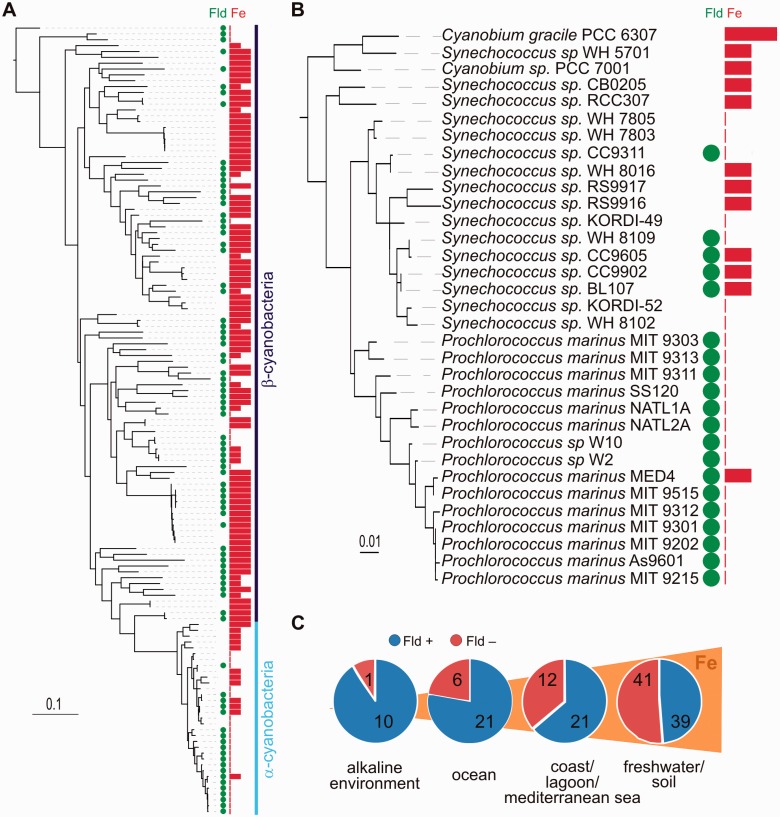
Environmental and phylogenetic distribution of Fld-coding genes among cyanobacteria. (*A*) Phylogenetic tree of cyanobacteria based on the 16S rRNA sequences. For each species, the presence (green circles) or absence of Fld, as well as the iron levels (red bars) at the site of isolation, is shown. Length of the red bars provides an estimation of iron level as high, intermediate, or low. The GEE statistical analysis, which takes into account the phylogenetic relationships of the organisms under study, shows a strong negative correlation (*P* < 10^−5^) between the presence of the Fld-coding gene and iron bioavailability. The tree was rooted using *Gloeobacter violaceous* sequence as outgroup. The scale bar indicates the number of substitutions per site. (*B*) The region of the phylogenetic tree corresponding to α-cyanobacteria is shown in the enlarged view. (*C*) Distribution of Fld-coding genes from cyanobacteria in different habitats. The presence (blue) or absence (purple) of Fld is represented by color codes, whereas the total numbers of isolates analyzed are indicated within each circle. The pie charts are only intended to be a visual aid as they do not show the phylogenetic relationships between isolates, and the numbers of isolates vary widely among habitats. The strains isolated from each habitat, their classification into α/β-cyanobacteria, and the presence/absence of the Fld-coding gene can be found in supplementary table S2, Supplementary Material online. Increasing iron levels are intended for illustration and do not represent actual concentrations.

To test whether similar selective pressures have impacted the genomes of coexisting photosynthetic prokaryotes, we repeated the analysis with cyanobacteria. A search for genes encoding Fld in the 191 isolates with sequenced genomes available at the IMG database (March 2014) retrieved sequences from 110 genomes. No Fld sequences were found in the remaining 81 genomes (8 out of the 81 were partial genomes and therefore not used for the study). In 32 completely sequenced genomes, the isolation location was not clear or unknown. The corresponding data were also excluded from the analysis (supplementary table S2, Supplementary Material online). The phylogenetic relationships between cyanobacteria were inferred by a maximum-likelihood tree from their 16S rRNA sequences, which are extensively used in phylogenetic analyses of prokaryotes (fig. 5*A*).

As in photosynthetic eukaryotes, a clear tendency toward Fld absence in iron-replete regions was observed in cyanobacteria, whereas Fld presence prevailed in iron-deficient habitats (fig. 5). Interestingly, all isolates of the genus *Prochlorococcus* were shown to contain Fld irrespective of the isolation site (fig. 5*B* and supplementary table S2, Supplementary Material online). The frequency of Fld-containing cyanobacteria (represented as the fraction of total cyanobacteria) increased with iron deficiency, from 49% (39/80) for brackish lakes/freshwater/soil locations to 64% (21/33) for marine coasts and mediterranean seas (Baltic, Mediterranean and Red seas), 78% (20/27) for the open ocean, and 91% (10/11) for alkaline environments (alkaline hot springs, alkaline lakes, limestones, and calcareous rocks). The GEE approach confirmed a negative correlation (*P* < 10^−^^5^) between iron bioavailability and the presence of Fld in cyanobacteria when considering the three categories of iron levels described above.

### Environmental Distribution of Fld-Coding Genes in the Most Abundant Marine Cyanobacteria Using Metagenomic Databases

We analyzed metagenomic data as a complementary approach to investigate the presence of Fld-coding genes in cyanobacterial populations thriving in different marine habitats. We computed the number of Fld hits in the available databases relative to those of selected single-copy core genes that are considered to be essential for survival (*recA*, *rpoB*, *gyrB*) and photosynthesis (*psbO*). Databases chosen for the analyses were the GOS metagenomes, as they are the largest coherent marine data set (Rusch et al. 2007).

**Fig. 6.—:**
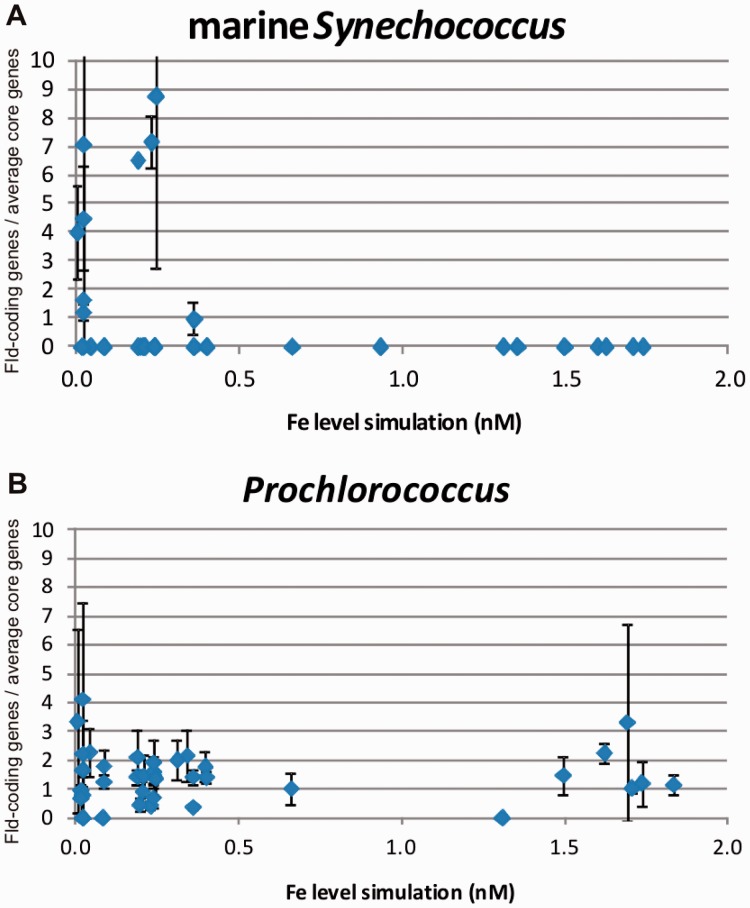
Correlation of Fld hits abundance recovered from GOS samples with predicted iron levels in marine *Synechococcus* (*A*) and *Prochlorococcus* (*B*). Details of the determinations are given under Materials and Methods. Iron values correspond to predicted concentrations published by Toulza et al. (2012).

Analyses were limited to α-cyanobacteria, specifically marine *Synechococcus* and *Prochlorococcus*, as they are virtually the only phototrophs represented in the GOS samplings. They are not only the most abundant phototrophs in marine habitats (and on Earth) but they are also enriched in these samples by the filtering process in which filamentous cyanobacteria and eukaryotes are excluded due to their higher size (Rusch et al. 2007). Previous studies of the GOS metagenomic data revealed that among surface oceanic prokaryotes, Fld presence showed a negative correlation with Fe abundance (Toulza et al. 2012). We used the published predicted values of iron at the sites where GOS samples were taken (Toulza et al. 2012) to analyze the iron-dependency of Fld distribution in marine *Synechococcus* and *Prochlorococcus*. We observed a negative correlation with iron bioavailability in marine *Synechococcus* (fig. 6*A*, Spearman Rho = −0.42, *P* = 0.007), whereas *Prochlorococcus* failed to display such a pattern (fig. 6*B*).

These results are in agreement with genomic analyses of these two groups of cyanobacteria (fig. 5*B*). Fld was present in all 13 completely sequenced genomes from *Prochlorococcus* but absent in 12 of 19 sequenced genomes of marine *Synechococcus* (fig. 5*B* and supplementary table S2, Supplementary Material online). Therefore, *Prochlorococcus* is an exception in which the gene encoding Fld appears to be a core gene, while still showing typical iron-responsive expression (Thompson et al. 2011). This is a particularly interesting case because this genus not only has a great ecological significance but also contains some members whose genomes are among the smallest within free-living organisms (Raven et al. 2013). The reasons for this unique feature of *Prochlorococci* are unknown. Additional research employing both genetic and/or pharmacological approaches will be required to evaluate the essentiality of the Fld-coding gene in these cyanobacteria.

Our results differ from those of Rivers et al. 2009, who reported that *Prochlorococcus*-like Fld sequences declined in coastal regions with respect to the open oceans, whereas *Synechococcus*-like genes exhibited the opposite trend. The reasons for this discrepancy might be related to different methodologies and queries used in both studies. Rivers et al. (2009) classified their samples into coastal and oceanic, and made a pool of raw data for each habitat, whereas we related the retrieved sequences with the iron level of the isolation site, excluding all those habitats in which the iron contents were not provided. The observation that the Fld-coding gene is a core gene in *Prochlorococcus* agrees with previous reports (Thompson et al. 2011; Kashtan et al. 2014), and suggests that the ratio of this gene to other core genes should be fairly constant for all habitats, as shown in figure 6*B*.

## Discussion

Oxygenic photosynthesis first evolved in the ancestors of modern cyanobacteria more than 2,500 Ma, and their light-harvesting capabilities were later exploited by eukaryotic cells already containing nucleus, cytoskeleton, and mitochondria through endosymbiosis, which turned a free-living microorganism into an organelle (Keeling 2013). The evidence collected so far on the origin of plastids indicates that successful endosymbiosis occurred only once to generate the so-called “primary” plastids in the common ancestor of green, red, and glaucophyte algae. All extant plastids appear to derive from this seminal event, either directly or through secondary and tertiary endosymbioses of green or red algae (supplementary fig. S2, Supplementary Material online).

**Fig. 7.—:**
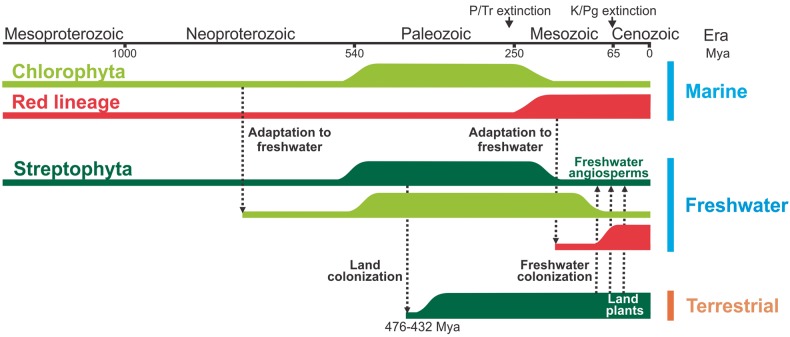
Schematic representation of the colonization of the main habitats by eukaryotic phototrophs and their diversification and variations in abundance during evolution. The heights of the horizontal bars indicate relative abundances according to Leliaert et al. (2011), Falkowski et al. (2004), and Becker and Marin (2009). The timeframes of the different events are approximate. The transition of streptophytes from terrestrial to freshwater habitats occurred several times during evolution. P/Tr extinction, End-Permian or Permian-Triassic mass extinction; K/Pg extinction, Cretaceous/Paleogene extinction (dinosaurs).

A schematic model describing the distribution of photosynthetic eukaryotes in different habitats is provided in figure 7. *Viridiplantae* split early (700–1,200 Ma) into *Chlorophyta* and *Streptophyta* (fig. 3), which followed radically different evolutionary paths (Leliaert et al. 2011). Chlorophytes radiated in marine and coastal environments and diversified to encompass a large variety of body forms, ecophysiological traits, and life cycle strategies (Leliaert et al. 2011, and references therein). They were dominant in the oceanic phytoplankton of the Paleozoic as evidenced by fossil records (O’Kelly 2007), being displaced during the Mesozoic by dinoflagellates, haptophytes, and diatoms (fig. 7) (Falkowski et al. 2004). Streptophytes, on the other hand, evolved largely in freshwater. Similar to the marine environments situation, dinoflagellates, diatoms, and golden algae (stramenopiles) gradually dominated freshwater habitats in the Early Cretaceous and Cenozoic (fig. 7) (Becker and Marin 2009). Streptophytes colonized dry lands approximately 450 Ma, giving rise to the *Embryophyta* (land plants) subclade (McCourt et al. 2004), which have dominated terrestrial environments ever since; some of them have even become secondarily adapted to aquatic habitats (fig. 7) (Chambers et al. 2008). Colonization of the firm land implied dealing with variable water availability and temperature, as well as increased exposure to radiation and absence of a buoyant medium. Among the numerous physiological and morphological resources that allowed the adaptation to terrestrial environments, some appear to be previous to the origin of land plants due to their presence in one or more members of the *Chlorophyta* (Leliaert et al. 2011). The list includes cellulosic cell walls, multicellularity, differentiated cells and tissues, plasmodesmata, zygote retention, and placenta (Leliaert et al. 2011). Further adaptations to dry land involved enhanced osmoregulation and osmoprotection, heat resistance, and desiccation and freezing tolerance, as well as the development of hormone-mediated signal transduction pathways required to direct and control plant responses to these environmental stimuli (Rensing et al. 2008; Leliaert et al. 2011). Primitive versions of some of these mechanisms are already found in the terrestrial charophyte *K. flaccidum* (Hori et al. 2014).

The phylogenetic tree inferred for Fld (fig. 1) revealed two unexpected features in the evolutionary history of this gene, relative to the origins of both the eukaryotic and the α-cyanobacterial Flds. It is generally assumed that genes which are only found in photosynthetic eukaryotes were already present in the genome of the original cyanobacterial endosymbiont, and were retained during diversification of algal taxa, either in the nuclear or in the plastid genome. Thus, phylogenetic trees of these genes are expected to have cyanobacteria in a basal place with respect to eukaryotes, which is indeed the case for typical photosynthetic products such as PsbA (Moore et al. 2008) and PsbO (fig. 2*B*). However, the topology of the long-chain Fld tree does not support this contention, as no cyanobacterial Fld sequence could be placed in a basal position respect to the eukaryotic Fld subclades (fig. 1*C*). Although this observation is still consistent with a single origin of the eukaryotic Fld-coding gene, it raises the intriguing possibility that this gene may be from a noncyanobacterial origin, and that Fld might have entered the eukaryotic domain by HGT after the primary endosymbiosis event. There are several reports of genes for plastid-targeted proteins which have been most likely acquired from noncyanobacterial prokaryotes (Keeling 2010). Alternatively, the HGT acceptor could have been the ancestral cyanobacterium that gave rise to modern plastids instead of an eukaryote (Gross et al. 2008). Available data do not permit to establish a definite origin for algal Fld, and a more extensive survey of Fld sequences will be necessary to properly address this issue.

The Fld tree topology (fig. 1), the common sequence signature shared by eukaryotes and α-cyanobacteria (supplementary fig. S3, Supplementary Material online), and the basal position occupied by algal Flds relative to their α-cyanobacterial counterparts (fig. 1*C*) also suggest that the Fld found in α-cyanobacteria was obtained through an HGT event from *Eukarya* to the *Synechococcus*/*Prochlorococcus* ancestor. In this sense, Fld differs from other photosynthetic proteins, which usually place eukaryotes closer to β-cyanobacteria, as illustrated for PsbO in figure 2*B*. It is worth mentioning that Flds from α- and β-cyanobacteria are phylogenetically distant (fig. 1*B*), even when cyanobacteria form a monophyletic group (Zhaxybayeva et al. 2006).

It has been proposed that the α-cyanobacterial lineage has a freshwater origin (see for instance Blank and Sánchez-Baracaldo 2010). Interestingly, early diverging α-cyanobacteria such as *Cyanobium gracile* PCC6307 (freshwater), *Cyanobium* sp. PCC7001 (intertidal zone), *Synechococcus* WH5701 (coastal), *Synechococcus* sp. CB0205 (estuary), and *Synechococcus* RCC307 (Mediterranean Sea) do not contain Fld (fig. 4*B*). The negative correlation between Fld presence and iron levels suggest that ancestral α-cyanobateria might have lacked this gene owing to their origin in an iron-replete environment. When they colonized marine environments, the reacquisition of Fld (presumably from an eukaryote), helped this lineage to tolerate temporal fluctuations in Fe levels, or even the chronic deficiencies typical of open oceans. Although this proposal is speculative, it is consistent with the adaptive nature of Fld in phototrophic organisms and the beneficial effects conferred by its expression against environmental and nutritional challenges (Tognetti et al. 2006, 2007). A number of observations also provide circumstantial support to this tenet. Fld is not the only photosynthetic protein of α-cyanobacteria to have been acquired through HGT. Actually, the main criterion for classification as α-cyanobacteria is to contain Form IA rubisco, which is more similar to the enzyme of proteobacteria than to the Form IB rubisco found in the other cyanobacteria (Rae et al. 2013). Moreover, a similar report and proposal, namely, HGT from an eukaryote to the ancestors of α-cyanobacteria has been made for the class I fructose bisphosphate aldolase gene (Rogers, Patron, et al. 2007). Elucidation of this intriguing feature of Fld evolution certainly deserves further investigation.

Within this context, HGT of an Fld-coding gene from α-cyanobacteria to the archaea *N. maritimus* (fig. 1*C*, see also Walker et al. 2010) is particularly significant as it would imply three consecutive interdomain transfer events: *Bacteria*→*Eukarya*→*Bacteria*→*Archaea*, highlighting the plasticity and adaptive value of Fld. Analysis of bacterial HGT events has shown that most transfers are indeed transient (Keeling and Palmer 2008) and that the kinds of genes that are found to be often transferred, such as those encoding Fld, usually confer short-term advantages linked to a particular habitat. As they are more prone to be transferred, they are more likely to be later discarded (Keeling and Palmer 2008).

In conclusion, the relative positions of eukaryotic and α-cyanobacterial Flds argue against a simple, lineal propagation of this trait. Rather, it appears that the Fld-coding gene has been lost and gained several times through HGT events involving the three domains of life, some of them taking place quite recently in an evolutionary scale.

Involvement of Fld in the response of photosynthetic microorganisms to iron starvation has been documented before (McKay et al. 1999; Toulza et al. 2012), focusing on specific groups of organisms. The vastly expanded sequence data currently available in public databases provide an opportunity to identify differences in Fld distribution among distinct habitats on a global scale. In the present research we have performed a systematic and in-depth analysis of all available Fld sequences from photosynthetic organisms, and were able to statistically substantiate a strong negative correlation between the presence of Fld in eukaryotic and prokaryotic phototrophs and the level of iron in the environment (figs. 4 and 5). The results support iron deficiency as a major selective pressure for the retention of the Fld-coding gene in phototrophs. This adaptive advantage must be especially relevant for marine organisms, as iron limitation is a frequent stress in open oceans. Although the main value of Fld under iron deficit is certainly the taking over of Fd functions as the major electron-distributing hub in chloroplasts and cyanobacteria, this substitution might also permit reallocation of the scarce available iron to other metal-dependent routes. Reports on the Fd-share of cellular iron vary from 30–40% in Fe-replete *Thalassiosira weissflogii* (Erdner and Anderson 1999) to 20–30% in leaves (Terry and Abadía 1986), in all cases a substantial fraction, indicating that Fd replacement by Fld may play a major role in the iron homeostasis of photosynthetic organisms.

Genes encoding Fd have been found in the genomes of all organisms displaying oxygenic photosynthesis, with no reported exception (Pierella Karlusich et al. 2014, and references therein). As iron availability is regarded as a main selective pressure which determines the fate of many genes in phototrophs (Rocap et al. 2003; Palenik et al. 2007), it is surprising that Fd was retained in spite of its susceptibility to iron starvation and oxidants, whereas Fld disappeared from the plant genome. Other Fe-containing proteins, such as cytochrome *c*_6_ and Fe-superoxide dismutase, have been totally or partially displaced by iron-free counterparts in the course of evolution (De la Rosa et al. 2006; Miller 2012). The reasons for Fd retention are likely related to its higher efficiency with respect to Fld in all photosynthesis-related reactions assayed so far, including photoreduction by PSI (Meimberg and Mühlenhoff 1999), and electron donation to Fd-NADP^+^ reductase (FNR), Fd-thioredoxin reductase (Tognetti et al. 2006), nitrite reductase, and glutamine synthetase (Vigara et al. 1998). FNR-mediated reactions are the best characterized at the kinetic level, revealing some interesting features. The *k*_cat_/*K*_M_ value of *Anabaena* Fd was reported to be approximately 25-fold higher than that of Fld (Medina et al. 1998). The strength of the interaction with *Anabaena* FNR, as reflected by the Michaelis constants, was similar for both carriers, indicating that the gain in efficiency was at the expense of turnover rates (Medina et al. 1998). It is likely that the complex electronic configuration of the flavin is not flexible enough to attain the high electron transfer rates typical of transition metals. Iron ions contain incompletely filled *d* orbitals which can readily accept electrons from different partners with various geometries, making them particularly versatile in oxido-reductive processes. It is indeed remarkable that some of these electron transfer reactions can be mimicked by the particular arrangement of π-orbitals found in the isoalloxazine ring system.

Photosynthetic electron transport is usually 1 order of magnitude faster than most heterotrophic oxidoreductive processes, and increases in catalytic efficiency have also been observed for FNR after recruitment into the photosynthetic electron transport chain (Carrillo and Ceccarelli 2003). Then, if Fd did confer the highest rates of electron distribution to photosynthetic acceptors under iron-replete conditions, it could still hold enough selective value to warrant retention in the genomes of phototrophs, including those which spend most of their lifetime in iron-deficient habitats. Indeed, available evidence indicates that photosynthetic organisms become nonviable below a certain threshold of Fd content (Holtgrefe et al. 2003; Mazouni et al. 2003), even in the presence of Fld (Poncelet et al. 1998; Blanco et al. 2011). The results suggest that Fld might help to alleviate the symptoms of iron limitation and other environmental hardships, but that some residual Fd activity is required to ensure survival and reproduction.

A few Fld-lacking cyanobacterial species have been found associated with iron-poor waters (fig. 5), indicating that Fld is probably the most important but not the only factor determining survival under iron deficit. Several other mechanisms are also put into action to overcome iron limitation, such as high surface-to-volume ratio to aid nutrient uptake (Chisholm 1992), a more extensive machinery for metal storage (Toulza et al. 2012), a decrease of iron-rich PSI (12 iron atoms per complex) in favor of PSII (Barber et al. 2006), the substitution of phycobilisomes by an iron-stress induced antenna (Ryan-Keogh et al. 2012), and extensive replacement of iron-dependent proteins (other than Fd) by isofunctional counterparts (De la Rosa et al. 2006; Miller 2012). Palenik et al. (2003) have described in some detail the strategies for iron conservation in *Synechococcus* sp. WH8102, an oceanic α-cyanobacterium living in iron-limited regions (see fig. 4*B*) which lacks not only Fld but also any detectable system for siderophore synthesis and uptake. These strategies include the use of Cu-containing plastocyanin for electron transport instead of cytochrome *c*_6_, as well as Ni-dependent superoxide dismutase and cobalt-dependent ribonucleotide reductase (Palenik et al. 2003).

Terrestrial plants have evolved from coastal/freshwater macroalgae belonging to the *Streptophyta* (charales, choleochaetales). Then, the loss of Fld in land plants could be associated with the passage of their algal ancestors from a marine habitat to coastal/freshwater and then terrestrial environments. We propose that these streptophytes, thriving in an environment in which iron was both abundant and readily accessible, had no need to induce Fld expression to replace Fd. Under such conditions, selection pressures for Fld retention as an adaptive trait may have been relaxed. Indeed, the geographical distribution of marine phototrophs shows that Fld-lacking algae and cyanobacteria are more represented in iron-rich coastal and freshwater regions (figs. 4 and 5 and supplementary fig. S5, Supplementary Material online), increasing the probability that the streptophyte precursor that gave origin to land plants already lacked the Fld-coding gene. Although this genetic asset could have disappeared from the plant genome between the first land-adapted streptophytes and the advent of vascular plants, both phylogenetic analyses and environmental distribution of Fld-containing and Fld-lacking organisms strongly support the notion that the Fld-coding gene was lost at an earlier stage, most likely during the transition between chlorophytes and streptophytes. In line with this assumption, Fld-coding sequences were not found in genomic or EST databases from the *Charophyta* (Timme et al. 2012; Hori et al. 2014), suggesting that the gene is absent from the whole clade.

After colonization of the firm land, plants faced a novel challenge: How to get iron from an environment in which the metal was plentiful but not readily available. Given that the Fld-coding gene might have been already absent in their genomes, they had to adapt to the new situation by other mechanisms. They accomplished that by way of a multigenic response in which many genes were recruited to operate at various levels, including rizhosphere acidification, reduction of Fe^+3^ to Fe^+2^, iron chelation and metal transport, all of them contributing to optimization of iron uptake (Tognetti et al. 2007). This strategy was successful enough to allow spreading of plant lineages throughout most of the planet. It is somehow surprising, however, that they were not able to recover an Fld-coding gene by, for instance, HGT from contemporary algae or bacteria. It should be borne in mind, however, that the evolution of land plants has been largely associated with expansion and diversification of existing gene families as the result of large-scale gene or whole-genome duplication events (Rensing et al. 2008), rather than incorporation of novel genes and functions (Bock 2010). In fact, examples of HGT to multicellular eukaryotes are rare (Rumpho et al. 2008; Bock 2010), and this limitation might have prevented recovery of Fld in spite of the beneficial effects still derived from its expression in plants (Tognetti et al. 2006, 2007; Coba de la Peña et al. 2010). Although this conjecture is consistent with current observations, experimental validation is lacking and further research will be required to properly address this issue.

## Supplementary Material

Supplementary text, figures S1–S5, and tables S1 and S2 are available at *Genome Biology and Evolution* online (http://www.gbe.oxfordjournals.org/).

Supplementary Data
