# Supplementary material for: Environmental Selection Pressures Related to Iron Utilization Are Involved in the Loss of the Flavodoxin Gene from the Plant Genome
Source: Genome Biol Evol. 2015 Feb 16;7(3):750–67. doi: 10.1093/gbe/evv031 (PMC5322553; doi:10.1093/gbe/evv031)
Supplement: Supplementary Data [file supp_7_3_750__index.html]

Environmental selection pressures related to iron utilization are involved in the loss of the flavodoxin gene from the plant genome — Environmental Selection Pressures Related to Iron Utilization Are Involved in the Loss of the Flavodoxin Gene from the Plant Genome — Supplementary Data 

# Environmental Selection Pressures Related to Iron Utilization Are Involved in the Loss of the Flavodoxin Gene from the Plant Genome

## Supplementary Data

files

**Files in this Data Supplement:**

- Supplementary Data - pdf file
- Supplementary Data - xls file
- Supplementary Data - xls file
- Supplementary Data - doc file
