# Supplementary material for: Environmental Selection Pressures Related to Iron Utilization Are Involved in the Loss of the Flavodoxin Gene from the Plant Genome
Source: Genome Biol Evol. 2015 Feb 16;7(3):750–67. doi: 10.1093/gbe/evv031 (PMC5322553; doi:10.1093/gbe/evv031)

Figure legends

Fig. S1: Structural superimposition of eleven diverse Flds. Crystal structures were obtained using the HHpred server over the PDB database using as a query the Fld from *Prochlorococcus marinus* MED4 [GenBank accession number CAE19630.1]. The sequence identity of the protein structures to the query ranged from 21% to 42%. The overall RMSD was <3.0 Å in an all-against-all comparison of the structures using the program Stamp, while the inter-structure sequence identity ranged from 21% to 69%. The similarly positioned ligands are displayed as sticks. One of the structures (1OBO) is depicted as cartoon colored from blue (Nter) to red (Cter). The remaining 10 structures (2WC1, 1YOB, 2FCR, 1CZN, 1AG9, 1F4P, 3F6R, 4HEQ, 2FZ5 and 5NUL) are shown as lines for clarity.

Fig. S2: Schematic representation of plastid evolution. A simplified plastid phylogenetic tree was constructed using data from Keeling 2010 and Leliaert et al. 2011. Tertiary and serial endosymbiosis events that occurred in some dinoflagellates (*Alveolata*) were omitted for simplicity.

Fig. S3: Sequence alignment of Flds from α-cyanobacteria, algae, the archaea *N. maritimus*, *E. coli* and some β-cyanobacteria. The squared region shows a conserved region present in the Flds from α-cyanobacteria, algae and the archaea *N. maritimus*. Numbers at the top of the alignment correspond to residue positions in the Fld from *Prochlorococcus marinus* MED4. Individual residues are colored according to ClustalX color coding.

Fig. S4: Phylogenetic tree of Fds and proteins with Fd-like domains. The tree topology indicates that Fds from cyanobacteria and plastids have a monophyletic origin. Grey branches correspond to Fds from non-photosynthetic microorganisms and to multi-domain proteins with Fd-like domains. The scale bar indicates the number of expected amino acid substitutions per site per unit of branch length.

Fig. S5: Geographical distribution of isolated algae in which the presence or absence of Fld has been confirmed. Each circle indicates an isolate containing (blue) or not (orange) Fld-coding genes. The map was redrawn using OpenStreetMap (http://www.openstreetmap.org).

Fig. S1

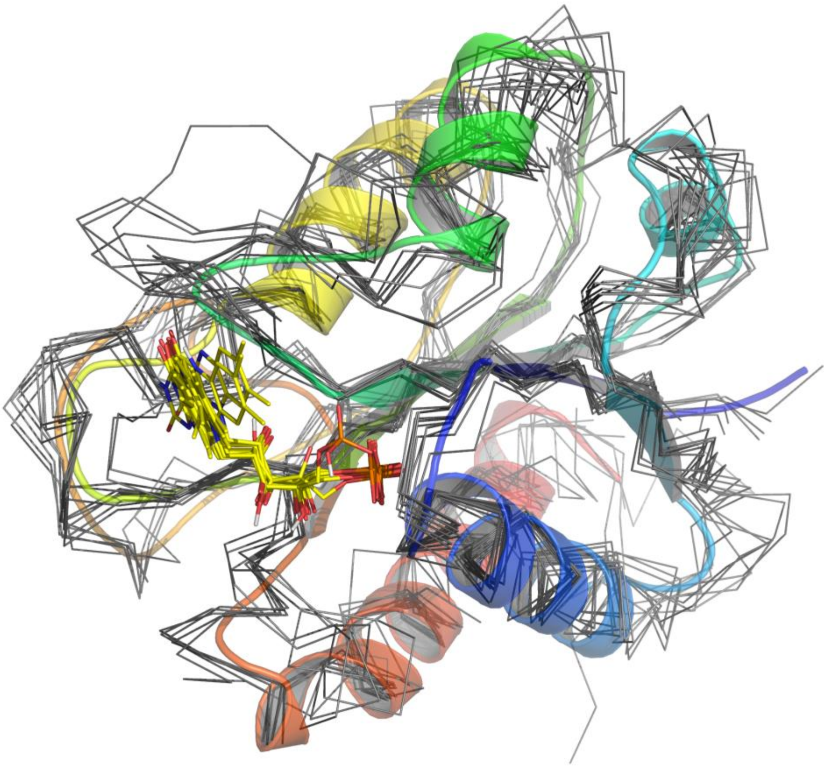

Fig. S2

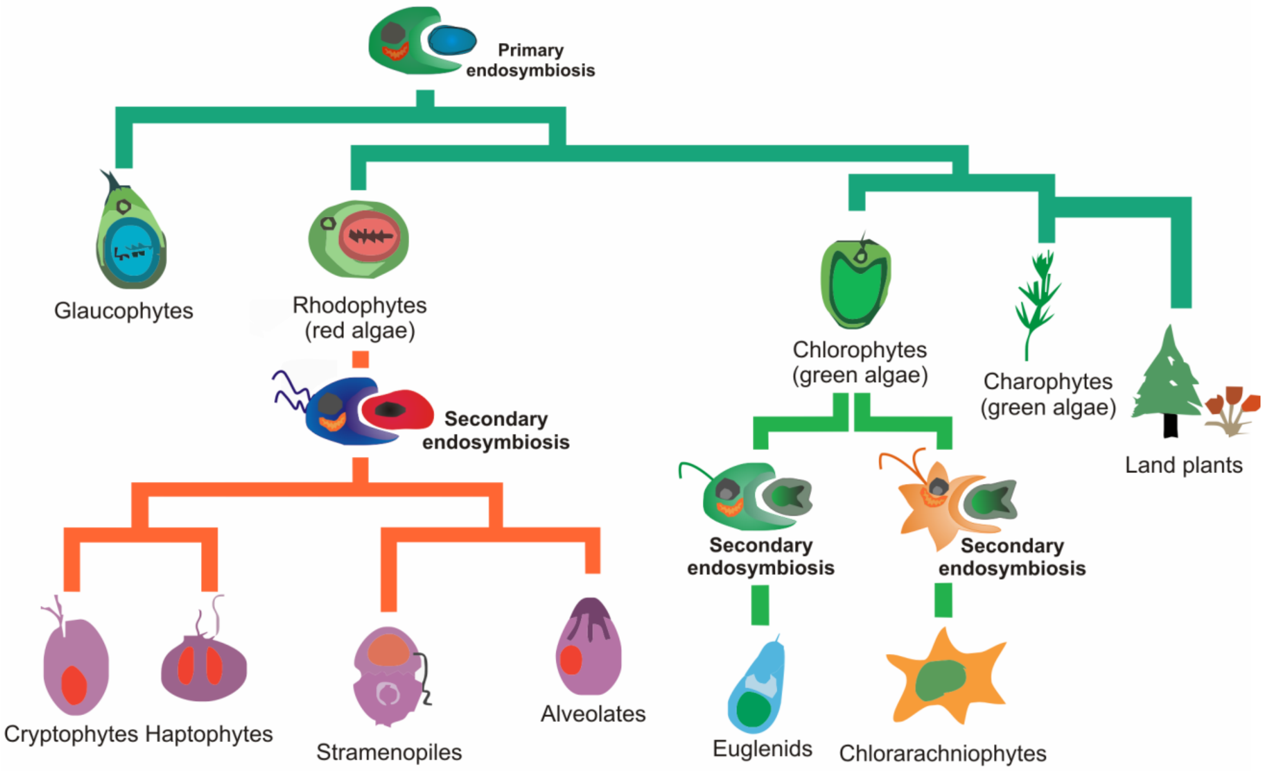
Primary endosymbiosis
Glaucophytes
Rhodophytes (red algae)
Secondary endosymbiosis
Chlorophytes (green algae)
Charophytes (green algae)
Land plants
Secondary endosymbiosis
Secondary endosymbiosis
Cryptophytes
Haptophytes
Stramenopiles
Alveolates
Euglenids
Chlorarachniophytes

Fig. S3

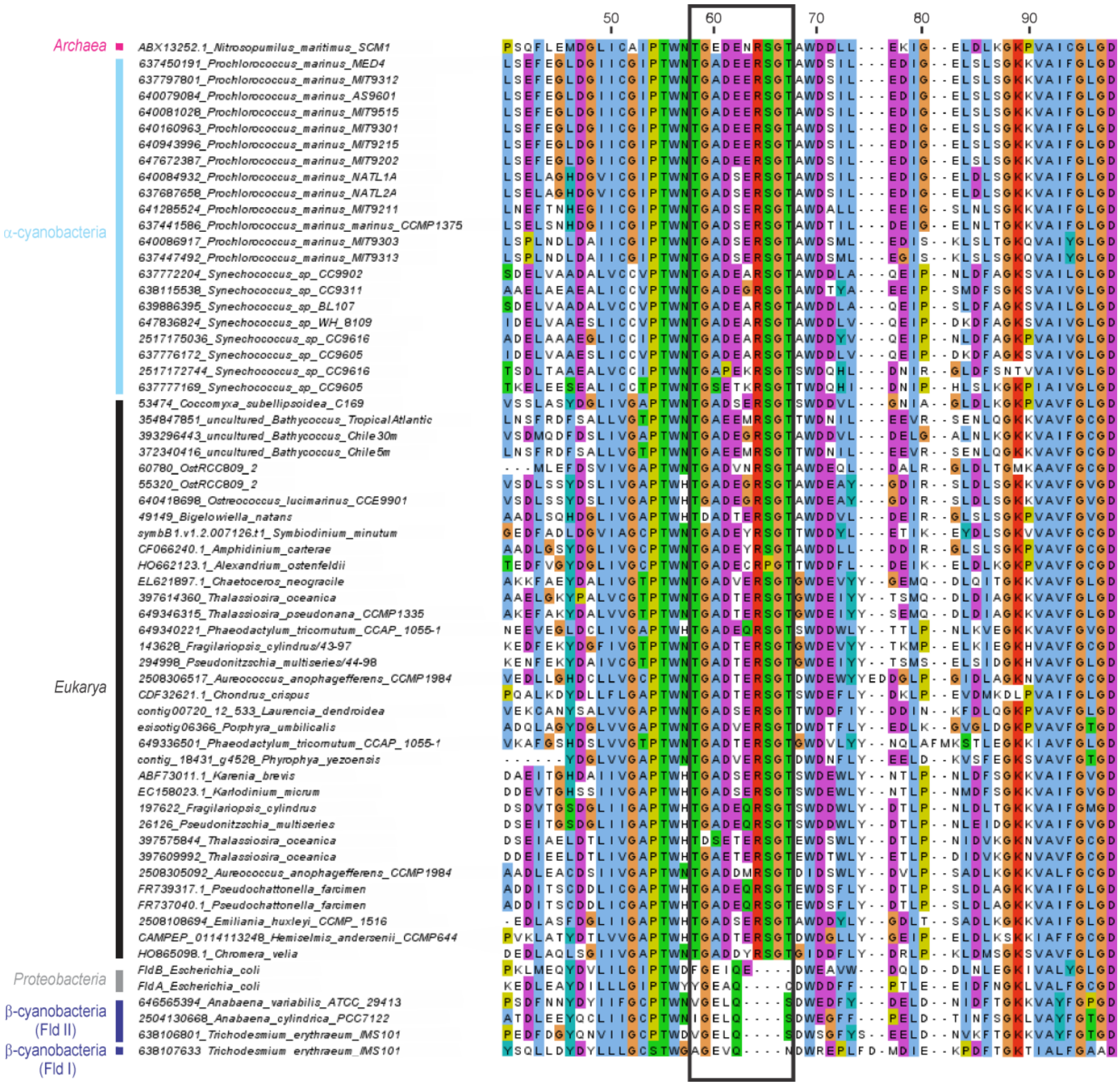
50
60
70
80
90
Archaea
α-cyanobacteria
Eukarya
Proteobacteria
β-cyanobacteria (Fld II)
β-cyanobacteria (Fld I)
ABX13252.1_Nitrosopumilus_maritimus_SCM1
637450191_Prochlorococcus_marinus_MED4
637797801_Prochlorococcus_marinus_MIT9312
640079084_Prochlorococcus_marinus_AS9601
640081028_Prochlorococcus_marinus_MIT9515
640160963_Prochlorococcus_marinus_MIT9301
640943996_Prochlorococcus_marinus_MIT9215
647672387_Prochlorococcus_marinus_MIT9202
640084932_Prochlorococcus_marinus_NATL1A
637687658_Prochlorococcus_marinus_NATL2A
641285524_Prochlorococcus_marinus_MIT9211
637441586_Prochlorococcus_marinus_marinus_CCMP1375
640086917_Prochlorococcus_marinus_MIT9303
637447492_Prochlorococcus_marinus_MIT9313
637772204_Synechococcus_sp_CC9902
638115538_Synechococcus_sp_CC9311
639886395_Synechococcus_sp_BL107
647836824_Synechococcus_sp_WH_8109
2517175036_Synechococcus_sp_CC9616
637776172_Synechococcus_sp_CC9605
2517172744_Synechococcus_sp_CC9616
637777169_Synechococcus_sp_CC9605
53474_Coccomyxa_subellipsoidea_C169
354847851_uncultured_Bathycoccus_TropicalAtlantic
393296443_uncultured_Bathycoccus_Chile30m
372340416_uncultured_Bathycoccus_Chile5m
60780_OstRCC809_2
55320_OstRCC809_2
640418698_Ostreococcus_lucimarinus_CCE9901
49149_Bigelowiella_natans
symbB1.v1.2.007126.t1_Symbiodinium_minutum
CF066240.1_Amphidinium_carterae
HO662123.1_Alexandrium_ostenfeldii
EL621897.1_Chaetoceros_neogracile
397614360_Thalassiosira_oceanica
649346315_Thalassiosira_pseudonana_CCMP1335
649340221_Phaeodactylum_tricornutum_CCAP_1055-1
143628_Fragilariopsis_cylindrus/43-97
294998_Pseudonitzschia_multiseries/44-98
2508306517_Aureococcus_anophagefferens_CCMP1984
CDF32621.1_Chondrus_crispus
contig00720_12_533_Laurencia_dendroidea
esisotig06366_Porphyra_umbilicalis
649336501_Phaeodactylum_tricornutum_CCAP_1055-1
contig_18431_g4528_Phyrophya_yezoensis
ABF73011.1_Karenia_brevis
EC158023.1_Karlodinium_micrum
197622_Fragilariopsis_cylindrus
26126_Pseudonitzschia_multiseries
397575844_Thalassiosira_oceanica
397609992_Thalassiosira_oceanica
2508305092_Aureococcus_anophagefferens_CCMP1984
FR739317.1_Pseudochattonella_farcimen
FR737040.1_Pseudochattonella_farcimen
2508108694_Emiliania_huxleyi_CCMP_1516
CAMPEP_0114113248_Hemiselmis_andersenii_CCMP644
HO865098.1_Chromera_velia
FldB_Escherichia_coli
FldA_Escherichia_coli
646565394_Anabaena_variabilis_ATCC_29413
2504130668_Anabaena_cylindrica_PCC7122
638106801_Trichodesmium_erythraeum_IMS101
638107633_Trichodesmium_erythraeum_IMS101

Fig. S4

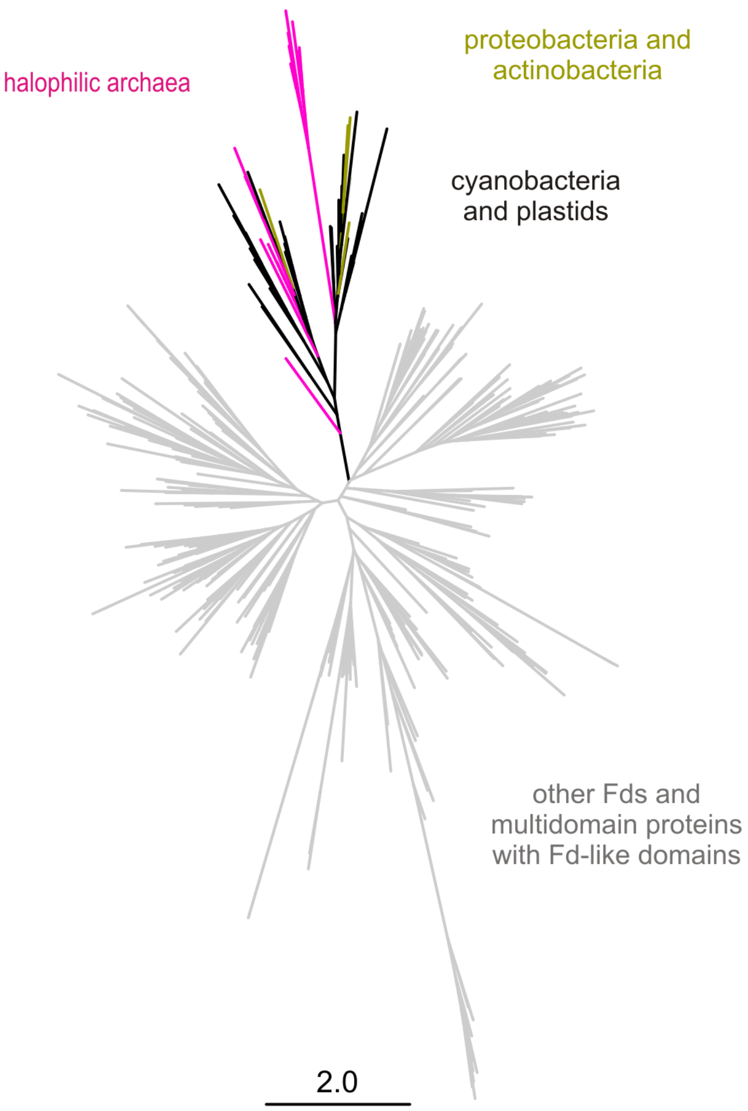

halophilic archaea
proteobacteria and
actinobacteria
cyanobacteria
and plastids
other Fds and
multidomain proteins
with Fd-like domains
2.0

Fig. S5

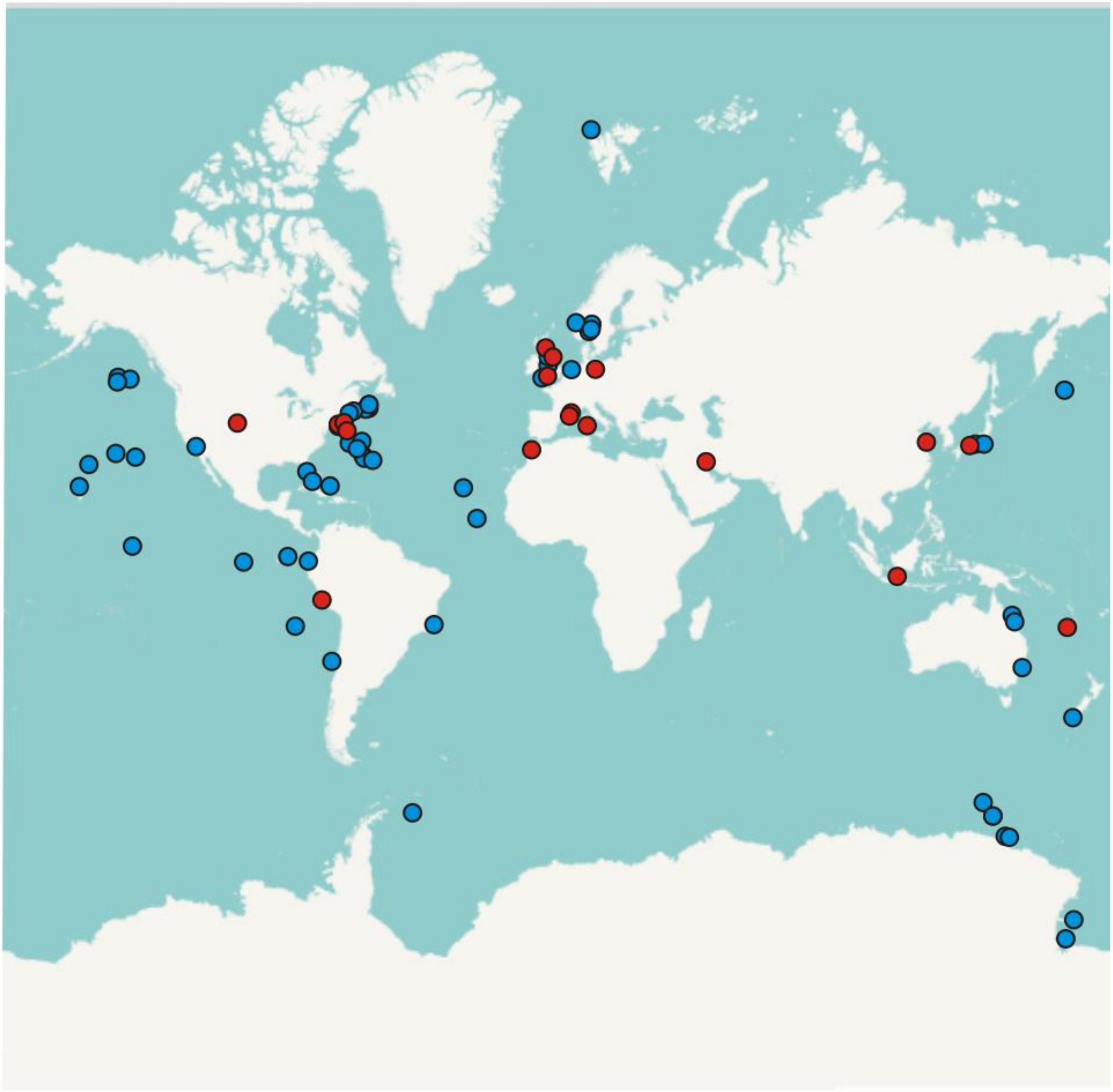

Supplement: Supplementary Data [file supp_evv031_SupplementaryData1_FiguresS1-S5.pdf]
