# Supplementary material for: Environmental Selection Pressures Related to Iron Utilization Are Involved in the Loss of the Flavodoxin Gene from the Plant Genome
Source: Genome Biol Evol. 2015 Feb 16;7(3):750–67. doi: 10.1093/gbe/evv031 (PMC5322553; doi:10.1093/gbe/evv031)
Supplement: Supplementary Data [file supp_evv031_SupplementaryData4_SuplementaryReferences.doc]

Abdel-Ghany SE, Müller-Moulé P, Niyogi KK, Pilon M, Shikanai T (2005) Two P-type ATPases are required for copper delivery in *Arabidopsis thaliana* chloroplasts. Plant Cell 17: 1233-1251.

Allen AE, Laroche J, Maheswari U, Lommer M, Schauer N, et al. (2008) Whole-cell response of the pennate diatom *Phaeodactylum tricornutum* to iron starvation. Proc Natl Acad Sci USA 105: 10438-10443.

Armbrust EV, Berges JA, Bowler C, Green BR, Martinez D, et al. (2004) The genome of the diatom *Thalassiosira pseudonana*: ecology, evolution, and metabolism. Science 306: 79-86.

Bandyopadhyay A, Elvitigala T, Welsh E, Stöckel J, Liberton M, et al. (2011) Novel metabolic attributes of the genus *Cyanothece*, comprising a group of unicellular nitrogen-fixing cyanobacteria. MBio 2: e00214-11.

Bench SR, Heller P, Frank I, Arciniega M, Shilova IN, Zehr JP (2013) Whole genome comparison of six Crocosphaera watsonii strains with differing phenotypes. J Phycol 49: 786-801.

Bench SR, Ilikchyan IN, Tripp HJ, Zehr JP (2011) Two strains of *Crocosphaera watsonii* with highly conserved genomes are distinguished by strain-specific features. FMICB 2.

Bhattacharya D, Price DC, Xin Chan C, Qiu H, Rose N, et al. (2013) Genome of the red alga *Porphyridium purpureum*. Nat Commun 4: 1941.

Bhaya D, Grossman AR, Steunou AS, Khuri N, Cohan FM, et al. (2007) Population level functional diversity in a microbial community revealed by comparative genomic and metagenomic analyses. ISME J 1: 703-713.

Blanc G, Duncan G, Agarkova I, Borodovsky M, Gurnon J, et al. (2010) The *Chlorella variabilis* NC64A genome reveals adaptation to photosymbiosis, coevolution with viruses, and cryptic sex. Plant Cell 22: 2943-2955.

Bogen C, Al-Dilaimi A, Albersmeier A, Wichmann J, Grundmann M, et al. (2013) Reconstruction of the lipid metabolism for the microalga *Monoraphidium neglectum* from its genome sequence reveals characteristics suitable for biofuel production. BMC Genomics 14: 926.

Carrieri D, Ananyev G, Lenz O, Bryant DA, Dismukes GC (2011) Contribution of a sodium ion gradient to energy conservation during fermentation in the cyanobacterium *Arthrospira* (*Spirulina*) *maxima* CS-328. Appl Environ Microbiol 77: 7185-7194.

Chan CX, Yang EC, Banerjee T, Yoon HS, Martone PT, et al. (2011) Red and green algal monophyly and extensive gene sharing found in a rich repertoire of red algal genes. Curr Biol 21: 328-333.

Chepurnov VA, Mann DG, Vyverman W, Sabbe K, Danielidis DB (2002) Sexual reproduction, mating system, and protoplast dynamics of *Seminavis* (Bacillariophyceae) J Phycol 38: 1004-1019.

Choi DH, Ryu JY, Kwon KK, Lee JH, Kim C, Lee CM, Noh JH (2013) Draft genome sequence of *Rubidibacter lacunae* strain KORDI 51-2T, a cyanobacterium isolated from seawater of Chuuk lagoon. Stand Genomic Sci 9: 197.

Cock JM, Sterck L, Rouzé P, Scornet D, Allen AE, et al. (2010) The *Ectocarpus* genome and the independent evolution of multicellularity in brown algae. Nature 465: 617-621.

Coleman ML, Sullivan MB, Martiny AC, Steglich C, Barry K, DeLong EF, Chisholm SW (2006) Genomic islands and the ecology and evolution of *Prochlorococcus*. Science, 311: 1768-1770.

Collén J, Porcel B, Carré W, Ball SG, Chaparro C, et al. (2013) Genome structure and metabolic features in the red seaweed *Chondrus crispus* shed light on evolution of the Archaeplastida. Proc Natl Acad Sci U S A 110: 5247-5252.

Curtis BA, Tanifuji G, Burki F, Gruber A, Irimia M, et al. (2012) Algal genomes reveal evolutionary mosaicism and the fate of nucleomorphs. Nature 492: 59-65.

Dagan T, Roettger M, Stucken K, Landan G, Koch R, et al. (2013) Genomes of Stigonematalean cyanobacteria (subsection V) and the evolution of oxygenic photosynthesis from prokaryotes to plastids. GBE 5: 31-44.

de Oliveira LS, Gregoracci GB, Silva GG, Salgado LT, Filho GA, et al. (2012) Transcriptomic analysis of the red seaweed *Laurencia dendroidea* (Florideophyceae, Rhodophyta) and its microbiome. BMC Genomics 13: 487.

Derelle E, Ferraz C, Rombauts S, Rouzé P, Worden AZ, et al. (2006) Genome analysis of the smallest free-living eukaryote *Ostreococcus tauri* unveils many unique features. Proc Natl Acad Sci U S A 103: 11647-11652.

DiTullio GR, Geesey ME, Maucher JM, Alm MB, Riseman SF, et al. (2005) Influence of iron on algal community composition and physiological status in the Peru upwelling system. Limnol Oceanogr 50: 1887-1907.

Doucette GJ, Erdner DL, Peleato ML, Hartman JJ, Anderson DM (1996) Quantitative analysis of iron-stress related proteins in *Thalassiosira weissflogii*: measurement of flavodoxin and ferredoxin using HPLC. Mar Ecol Prog Ser 130: 260-276.

Dufresne A, Ostrowski M, Scanlan DJ, Garczarek L, Mazard S, et al. (2008) Unraveling the genomic mosaic of a ubiquitous genus of marine cyanobacteria. Genome Biol: 9: R90.

Dufresne A, Salanoubat M, Partensky F, Artiguenave F, Axmann IM, et al. (2003) Genome sequence of the cyanobacterium *Prochlorococcus marinus* SS120, a nearly minimal oxyphototrophic genome. Proc Natl Acad Sci U S A 100: 10020-10025.

Erdner DL, Price NM, Doucette GJ, Peleato ML, Anderson DM (1999) Characterization of ferredoxin and flavodoxin as markers of iron limitation in marine phytoplankton. Mar Ecol Prog Ser 184: 43-53.

Everroad RC, Woebken D, Singer SW, Burow LC, Kyrpides N, et al. (2013) Draft genome sequence of an oscillatorian cyanobacterium, strain ESFC-1. Genome Announc 1: e00527-13.

Fiore MF, Alvarenga DO, Varani AM, Hoff-Risseti C, Crespim E, et al. (2013) Draft genome sequence of the Brazilian toxic bloom-forming cyanobacterium *Microcystis aeruginosa* strain SPC777. Genome Announc 1: e00547-13.

Fitzgerald MP, Husain A, Rogers LJ (1978) A constitutive flavodoxin from a eukaryotic alga. Biochem Biophys Res Commun 81: 630-635.

Fujisawa T, Narikawa R, Okamoto S, Ehira S, Yoshimura H, et al. (2010) Genomic structure of an economically important cyanobacterium, *Arthrospira* (*Spirulina*) *platensis* NIES-39. DNA Res: dsq004.

Gillard J, Devos V, Huysman MJJ, de Veylder L, D’Hondt S, et al. (2008) Physiological and transcriptomic evidence for a close coupling between chloroplast ontogeny and cell cycle progression in the pennate diatom *Seminavis robusta*. Plant Physiol 148: 1394-1411.

Gobler CJ, Berry DL, Dyhrman ST, Wilhelm SW, Salamov A, et al. (2011) Niche of harmful alga *Aureococcus anophagefferens* revealed through ecogenomics. Proc Natl Acad Sci U S A 108: 4352-4357.

Gordon RM, Johnson KS, Coale KH (1998) The behaviour of iron and other trace elements during the IronEx-I and PlumEx experiments in the Equatorial Pacific. Deep-Sea Res II 45: 995-1041.

Hori K, Maruyama F, Fujisawa T, Togashi T, Yamamoto N, et al. (2014) *Klebsormidium flaccidum* genome reveals primary factors for plant terrestrial adaptation. Nat Commun 5: 3978.

Inda LA, Peleato ML (2002) Immunoquantification of flavodoxin and ferredoxin from *Scenedesmus vacuolatus* (Chlorophyta) as iron-stress molecular markers. Eur J Phycol 37: 579-586.

Inda LA, Peleato ML (2003) Development of an ELISA approach for the determination of flavodoxin and ferredoxin as markers of iron deficiency in phytoplankton. Phytochemistry 63: 303-308.

Jaeckisch N, Yang I, Wohlrab S, Glöckner G, Kroymann J, et al. (2011) Comparative genomic and transcriptomic characterization of the toxigenic marine dinoflagellate *Alexandrium ostenfeldii*. PLoS One 6: e28012.

Jung G, Lee CG, Kang SH, Jin E (2007) Annotation and expression profile analysis of cDNas from the Antarctic diatom *Chaetoceros neogracile*. J Microbiol Biotechnol 17: 1330-1337.

Kaneko T, Nakajima N, Okamoto S, Suzuki I, Tanabe Y, et al. (2007) Complete genomic structure of the bloom-forming toxic cyanobacterium *Microcystis aeruginosa* NIES-843. DNA Res 14: 247-256.

Kettler GC, Martiny AC, Huang K, Zucker J, Coleman M L, et al. (2007) Patterns and implications of gene gain and loss in the evolution of *Prochlorococcus*. PLoS Genet 3: e231.

La Roche J, Murray H, Orellana O, Newton J (1995) Flavodoxin expression as an indicator of iron limitation in marine diatoms. J Phycol 31: 520-553.

Lane CE, van den Heuvel K, Kozera C, Curtis BA, Parsons BJ, et al. (2007) Nucleomorph genome of *Hemiselmis andersenii* reveals complete intron loss and compaction as a driver of protein structure and function. Proc Natl Acad Sci U S A 104: 19908-19913.

LaRoche J, Boyd PW, McKay RML, Geider RJ (1996) Flavodoxin as an *in situ* marker for iron stress in phytoplankton. Nature 382: 802-805.

LaRoche J, Geider RJ, Graziano LM, Murray H, Lewis K (1993) Induction of specific proteins in eukaryotic algae grown under iron-, phosphorus-, or nitrogen-deficient conditions. J Phycol 29: 767-777.

Li X, Yakunin AF, McKay RML (2004) Fe-responsive accumulation of redox proteins ferredoxin and flavodoxin in a marine cryptomonad. Eur J Phycol 39: 73-82.

Lommer M, Specht M, Roy AS, Kraemer L, Andreson R, et al. (2012) Genome and low-iron response of an oceanic diatom adapted to chronic iron limitation. Genome Biol 13: R66.

Malmstrom RR, Rodrigue S, Huang KH, Kelly L, Kern SE, et al. (2012). Ecology of uncultured *Prochlorococcus* clades revealed through single-cell genomics and biogeographic analysis. ISME J 7: 184-198.

Marsan D, Wommack KE, Ravel J, Chen F (2014) Draft genome sequence of *Synechococcus sp*. strain CB0101, isolated from the Chesapeake Bay estuary. Genome Announc 2: e01111-13.

Matsuzaki M, Misumi O, Shin-i T, Maruyama S, Takahara M, et al. (2004) Genome sequence of the ultrasmall unicellular red alga *Cyanidioschyzon merolae* 10D. Nature 428: 653-657.

Maucher JM, DiTullio GR (2003) Flavodoxin as a diagnostic indicator of chronic iron limitation in the Ross Sea and New Zealand sector of the Southern Ocean. In: Ditullio GR, Dunbar BR, editors. Biogeochemistry of the Ross Sea. Washington: American Geophysical Union.

McKay RML, Bullerjahn GS, Porta D, Brown RT, Sherrell RM, et al. (2004) Consideration of the bioavailability of iron in the North American Great Lakes: Development of novel approaches toward understanding iron biogeochemistry. Aquatic Ecosystem Health and Management 7: 475-490.

McKay RML, LaRoche J, Yakunin AF, Durnford DG, Geider RJ (1999) Accumulation of ferredoxin and flavodoxin in a marine diatom in response to Fe. J Phycol 35: 510-519.

McKay RML, Villareal TA, LaRoche J (2000) Vertical migration by *Rhizosolenia* spp*.* (Bacillariophyceae): implications for Fe acquisition. J Phycol 36: 669-674.

McMinn A, Hegseth EN (2004) Quantum yield and photosynthetic parameters of marine microalgae from the southern Arctic Ocean, Svalbard. J Mar Biol Ass UK 84: 865-871.

Merchant SS, Prochnik SE, Vallon O, Harris EH, Karpowicz SJ, et al. (2007) The *Chlamydomonas* genome reveals the evolution of key animal and plant functions. Science 318: 245-250.

Miller SR, Wood AM, Blankenship RE, Kim M, Ferriera S (2011) Dynamics of gene duplication in the genomes of chlorophyll d-producing cyanobacteria: implications for the ecological niche. GBE 3: 601-613.

Monier A, Welsh RM, Gentemann C, Weinstock G, Sodergren E, et al. (2012) Phosphate transporters in marine phytoplankton and their viruses: cross-domain commonalities in viral-host gene exchanges. Environ Microbiol 14: 162-176.

Moore RB, Oborník M, Janouskovec J, Chrudimský T, Vancová M, et al. (2008) A photosynthetic alveolate closely related to apicomplexan parasites. Nature 451: 959-963.

Moreau H, Verhelst B, Couloux A, Derelle E, Rombauts S, et al. (2012) Gene functionalities and genome structure in *Bathycoccus prasinos* reflect cellular specializations at the base of the green lineage. Genome Biol 13: R74.

Nakamura Y, Kaneko T, Sato S, Mimuro M, Miyashita H, et al. (2003) Complete genome structure of *Gloeobacter violaceus* PCC 7421, a cyanobacterium that lacks thylakoids (supplement). DNA Res 10: 181.

Nakamura Y, Sasaki N, Kobayashi M, Ojima N, Yasuike M, et al. (2013) The first symbiont-free genome sequence of marine red alga, Susabi-nori (*Pyropia yezoensis*). PLoS One 8: e57122.

Nelson DR, Mengistu S, Ranum P, Celio G, Mashek M, et al. (2013) New lipid-producing, cold-tolerant yellow-green alga isolated from the Rocky Mountains of Colorado. Biotechnol Prog 29: 853-861.

Nosenko T, Lidie KL, van Dolah FM, Lindquist E, Cheng JF, et al. (2006) Chimeric plastid proteome in the Florida "red tide" dinoflagellate *Karenia brevis*. Mol Biol Evol 23: 2026-2038.

O'Brien EA, Koski LB, Zhang Y, Yang L, Wang E, et al. (2007) TBestDB: a taxonomically broad database of expressed sequence tags (ESTs). Nucleic Acids Res 35: D445-451.

Palenik B, Brahamsha B, Larimer FW, Land M, Hauser L, et al. (2003) The genome of a motile marine *Synechococcus*. Nature 424: 1037-1042.

Palenik B, Grimwood J, Aerts A, Rouzé P, Salamov A, et al. (2007) The tiny eukaryote *Ostreococcus* provides genomic insights into the paradox of plankton speciation. Proc Natl Acad Sci USA 104: 7705-7710.

Palenik B, Ren Q, Dupont CL, Myers GS, Heidelberg JF, et al. (2006) Genome sequence of *Synechococcus* CC9311: insights into adaptation to a coastal environment. Proc Natl Acad Sci U S A 103: 13555-13559.

Pankowski A, McMinn A (2008) Ferredoxin and flavodoxin in eastern Antarctica pack ice. Polar Biol 31: 1153-1165.

Pankowski A, McMinn A (2009) Development of immunoassays for the iron-regulated proteins ferredoxin and flavodoxin in polar microalgae. J Phycol 45: 771-783.

Pankowski A, McMinn A (2009) Iron availability regulates growth, photosynthesis, and production of ferredoxin and flavodoxin in Antarctic sea ice diatoms. Aquat Biol 4: 273-288.

Patron NJ, Waller RF, Keeling PJ (2006) A tertiary plastid uses genes from two endosymbionts. J Mol Biol 357: 1373-1382.

Peleato ML, Ayora S, Inda LA, Gómez-Moreno C (1994) Isolation and characterization of two different flavodoxins from the eukaryote *Chlorella fusca*. Biochem J 302: 807-811.

Price DC, Chan CX, Yoon HS, Yang EC, Qiu H, et al. (2012) *Cyanophora paradoxa* genome elucidates origin of photosynthesis in algae and plants. Science 335: 843-847.

Price NT, Smith AJ, Rogers LJ (1991) Isolation of flavodoxin isoforms from a macroalga *Porphyra umbilicalis* and a conformational change on dissociation of flavin. Phytochemistry 30: 2835-2839.

Prochnik SE, Umen J, Nedelcu AM, Hallmann A, Miller SM, et al. (2010) Genomic analysis of organismal complexity in the multicellular green alga *Volvox carteri*. Science 329: 223-226.

Qiu H, Price DC, Weber AP, Reeb V, Yang EC, et al. (2013) Adaptation through horizontal gene transfer in the cryptoendolithic red alga *Galdieria phlegrea*. Curr Biol 23: R865-866.

Radakovits R, Jinkerson RE, Fuerstenberg SI, Tae H, Settlage RE, et al. (2012) Draft genome sequence and genetic transformation of the oleaginous alga *Nannochloropis gaditana*. Nat Commun 3: 686.

Ran L, Larsson J, Vigil-Stenman T, Nylander JA, Ininbergs K, Zheng WW, et al. (2010) Genome erosion in a nitrogen-fixing vertically transmitted endosymbiotic multicellular cyanobacterium. PLoS One 5: e11486.

Read BA, Kegel J, Klute MJ, Kuo A, Lefebvre SC, et al. (2013) Pan genome of the phytoplankton *Emiliania* underpins its global distribution. Nature 499: 209-213.

Rocap G, Larimer FW, Lamerdin J, Malfatti S, Chain P, et al. (2003) Genome divergence in two *Prochlorococcus* ecotypes reflects oceanic niche differentiation. Nature 424: 1042-1047.

Rogers LJ, Sykes GA (1990) Conformational changes in *Chondrus crispus* flavodoxin on dissociation of FMN and reconstitution with flavin analogues. Biochem J 272: 775-779.

Roy EG, Wells ML (2010) Evidence for regulation of Fe(II) oxidation by organic complexing ligands in the Eastern Subarctic Pacific. Marine Chem 127: 115-122.

Russell RB, Barton GJ (1992) Multiple protein sequence alignment from tertiary structure comparison: assignment of global and residue confidence levels. Proteins 14: 309-323.

Schönknecht G, Chen WH, Ternes CM, Barbier GG, Shrestha RP, et al. (2013) Gene transfer from bacteria and archaea facilitated evolution of an extremophilic eukaryote. Science 339: 1207-1210.

Shih PM, Wu D, Latifi A, Axen SD, Fewer DP, et al. (2013) Improving the coverage of the cyanobacterial phylum using diversity-driven genome sequencing. Proc Natl Acad Sci U S A 110: 1053-1058.

Six C, Finkel ZV, Rodriguez F, Marie D, Partensky F, et al. (2008) Contrasting photoacclimation costs in ecotypes of the marine eukaryotic picoplankter *Ostreococcus*. Limnol Oceanogr 53: 255–265.

Soding J, Biegert A, Lupas AN (2005) The HHpred interactive server for protein homology detection and structure prediction. Nucleic Acids Res 33: W244-248.

Starkenburg SR, Reitenga KG, Freitas T, Johnson S, Chain PS, Garcia-Pichel F, Kuske CR (2011) Genome of the Cyanobacterium *Microcoleus vaginatus* FGP-2, a Photosynthetic Ecosystem Engineer of Arid Land Soil Biocrusts Worldwide. J Bacteriol 193: 4569-4570.

Strzepek RF, Harrison PJ (2004) Photosynthetic architecture differs in coastal and oceanic diatoms. Nature 431: 689-692.

Stucken K, John U, Cembella A, Murillo AA, Soto-Liebe K, et al. (2010). The smallest known genomes of multicellular and toxic cyanobacteria: comparison, minimal gene sets for linked traits and the evolutionary implications. PLoS One 5: e9235.

Sugita C, Ogata K, Shikata M, Jikuya H, Takano J, et al. (2007). Complete nucleotide sequence of the freshwater unicellular cyanobacterium *Synechococcus elongatus* PCC 6301 chromosome: gene content and organization. Photosynthesis res 93: 55-67.

Swingley WD, Chen M, Cheung PC, Conrad AL, Dejesa LC, et al. (2008) Niche adaptation and genome expansion in the chlorophyll d-producing cyanobacterium *Acaryochloris marina*. Proc Natl Acad Sci U S A 105: 2005-2010.

Thiel T, Pratte BS, Zhong J, Goodwin L, Copeland A, et al. (2014) Complete genome sequence of *Anabaena variabilis* ATCC 29413. SIGS 9.

Tooming-Klunderud A, Sogge H, Rounge TB, Nederbragt AJ, Lagesen K, Glöckner G, et al. (2013) From green to red: Horizontal gene transfer of the Phycoerythrin gene cluster between Planktothrix strains. Appl Environ Microbiol 79: 6803-6812.

Trautmann D, Voß B, Wilde A, Al-Babili S, Hess WR (2012) Microevolution in cyanobacteria: re-sequencing a motile substrain of *Synechocystis sp*. PCC 6803. DNA Res: dss024.

Tripp HJ, Bench SR, Turk KA, Foster RA, Desany BA, Niazi F, et al. (2010) Metabolic streamlining in an open-ocean nitrogen-fixing cyanobacterium. Nature 464: 90-94.

Vaulot D, Lepère C, Toulza E, De la Iglesia R, Poulain J, et al. (2012) Metagenomes of the picoalga *Bathycoccus* from the Chile coastal upwelling. PLoS One 7: e39648.

Vieler A, Wu G, Tsai CH, Bullard B, Cornish AJ, et al. (2012) Genome, functional gene annotation, and nuclear transformation of the heterokont oleaginous alga Nannochloropsis oceanica CCMP1779. PLoS Genet 8: e1003064.

Villareal TA, McKay RML, Al-Rshaidat MMD, Boyanapalli R, Sherrell RM (2007) Compositional and fluorescence characteristics of the giant diatom *Ethmodiscus* along a 3000 km transect (28°N) in the central North Pacific gyre. Deep-Sea Res I 54: 1273-1288.

Voß B, Bolhuis H, Fewer DP, Kopf M, Möke F, et al. (2013) Insights into the physiology and ecology of the brackish-water-adapted cyanobacterium *Nodularia spumigena* CCY9414 based on a genome-transcriptome analysis. PloS one 8: e60224.

Wakabayashi S, Kimura T, Fukuyama K, Matsubara H, Rogers LJ (1989) The amino acid sequence of a flavodoxin from the eukaryotic red alga Chondrus crispus. Biochem J 263: 981-984.

Wang D, Ning K, Li J, Hu J, Han D, et al. (2014) Nannochloropsis genomes reveal evolution of microalgal oleaginous traits. PLoS Genet 10: e1004094.

Welsh EA, Liberton M, Stöckel J, Loh T, Elvitigala T, et al. (2008) The genome of *Cyanothece* 51142, a unicellular diazotrophic cyanobacterium important in the marine nitrogen cycle. Proc Natl Acad Sci U S A 105: 15094-15099.

Whitney LP, Lins JJ, Hughes MP, Wells ML, Chappell PD, et al. (2011) Characterization of putative iron responsive genes as species-specific indicators of iron stress in thalassiosiroid diatoms. Front Microbiol 2: e234.

Woehle C, Dagan T, Martin WF, Gould SB (2011) Red and problematic green phylogenetic signals among thousands of nuclear genes from the photosynthetic and apicomplexa-related *Chromera velia*. Genome Biol Evol 3: 1220-1230.

Worden AZ, Lee JH, Mock T, Rouzé P, Simmons MP, et al. (2009) Green evolution and dynamic adaptations revealed by genomes of the marine picoeukaryotes *Micromonas*. Science 324: 268-272.

Zumft WG, Spiller H (1971) Characterization of a flavodoxin from the green alga *Chlorella*. Biochem Biophys Res Commun 45: 112-118.
